# *TOPOISOMERASE 6B* is involved in chromatin remodelling associated with control of carbon partitioning into secondary metabolites and cell walls, and epidermal morphogenesis in *Arabidopsis*

**DOI:** 10.1093/jxb/eru198

**Published:** 2014-05-12

**Authors:** Amandeep Mittal, Rajagopal Balasubramanian, Jin Cao, Prabhjeet Singh, Senthil Subramanian, Glenn Hicks, Eugene A. Nothnagel, Noureddine Abidi, Jaroslav Janda, David W. Galbraith, Christopher D. Rock

**Affiliations:** ^1^Department of Biological Sciences, Texas Tech University, Lubbock, TX 79409-3131, USA; ^2^Tamil Nadu Agricultural University, Department of Plant Breeding and Genetics, Agricultural College and Research Institute, Madurai-625 104, India; ^3^Department of Biotechnology, Guru Nanak Dev University, Amritsar-143 005, Punjab, India; ^4^South Dakota State University, Department of Plant Science, Brookings, SD 57007, USA; ^5^Institute for Integrative Genome Biology, University of California, Riverside, CA 92521, USA; ^6^Department of Botany and Plant Sciences, University of California, Riverside CA 92521-0124, USA; ^7^Texas Tech University, Department of Plant and Soil Science and Fiber and Biopolymer Research Institute, 1001 East Loop 289, Lubbock, TX 79409-5019, USA; ^8^University of Arizona, Department of Plant Sciences and BIO5 Institute, 341 Keating Bldg, Tucson, AZ 85721, USA

**Keywords:** Anthocyanin, cell wall, endoreduplication, hypocotyl, light, oxidative stress, root hairless, skotomorphogenesis, starch.

## Abstract

Plant growth is continuous and modular, a combination that allows morphogenesis by cell division and elongation and serves to facilitate adaptation to changing environments. The pleiotropic phenotypes of the *harlequin* (*hlq*) mutant, isolated on the basis of ectopic expression of the abscisic acid (ABA)- and auxin-inducible *proDc3:GUS* reporter gene, were previously characterized. Mutants are skotomorphogenic, have deformed and collapsed epidermal cells which accumulate callose and starch, cell walls abundant in pectins and cell wall proteins, and abnormal and reduced root hairs and leaf trichomes. *hlq* and two additional alleles that vary in their phenotypic severity of starch accumulation in the light and dark have been isolated, and it is shown that they are alleles of *bin3/hyp6/rhl3*/Topoisomerase6B. Mutants and inhibitors affecting the cell wall phenocopy several of the traits displayed in *hlq*. A microarray analysis was performed, and coordinated expression of physically adjacent pairs/sets of genes was observed in *hlq*, suggesting a direct effect on chromatin. Histones, WRKY and IAA/AUX transcription factors, aquaporins, and components of ubiquitin-E3-ligase-mediated proteolysis, and ABA or biotic stress response markers as well as proteins involved in cellular processes affecting carbon partitioning into secondary metabolites were also identified. A comparative analysis was performed of the *hlq* transcriptome with other previously published TopoVI mutant transcriptomes, namely *bin3*, *bin5*, and *caa39* mutants, and limited concordance between data sets was found, suggesting indirect or genotype-specific effects. The results shed light on the molecular mechanisms underlying the *det/cop/fus*-like pleiotropic phenotypes of *hlq* and support a broader role for TopoVI regulation of chromatin remodelling to mediate development in response to environmental and hormonal signals.

## Summary

The *harlequin* mutant encodes subunit B of plant-specific topoisomerase VI. Transcriptomics showed synergistic effects on expression of adjacent genes, suggesting a broader role in chromatin remodelling during development and stress responses.

## Introduction

Plants are sessile and have evolved an open pattern of growth and development to give plasticity to their responses to changing environments. A central paradox of plant biology is that there exist only a handful of compounds (phytohormones) that mediate many complex processes in plant growth. A related conundrum is that, despite the elucidation of hormone structures, their biosynthetic pathways, and cognate hormone receptors, an understanding of the mechanisms of hormone action and ‘sensitivity’ remains elusive. Abscisic acid (ABA) is a plant hormone that mediates a myriad of physiological processes in growth and development, including cell division, water use efficiency, and gene expression during seed development and in response to environmental stresses, such as drought, chilling, salt, pathogen attack, UV, and high light (Rock *et al.*, 2010). It was previously shown that the *uidA* (β-glucuronidase; GUS) reporter, under the transcriptional regulation of the carrot (*Daucus carota*) *Late Embryogenesis-Abundant Dc3* promoter in transgenic *Arabidopsis thaliana* seedlings, is ABA and auxin inducible within the root zone of elongation and within the vasculature, and inducible in guard cells by ABA (Chak *et al.*, 2000; Rock and Sun, 2005). The *abi1-1* and *abi2-1* mutations reduce ABA-inducible *proDc3*:*GUS* expression in root tissues and interact with (attenuate) auxin signalling leading to *proDc3:GUS* expression. Furthermore, the *aux1* and *axr4* mutants, which are affected in auxin transport/homeostasis, show a hypomorphic effect on ABA-inducible *proDc3:GUS* expression, demonstrating that ABA and IAA signalling pathways interact in roots (Rock and Sun, 2005).

Several reports have established links between ABA and the cell wall. For example, ABA-dependent callose accumulation is required for β-aminobutyric acid-induced resistance to the pathogens *Alternaria brassicola* and *Plectosphaerella cumumerina* (Ton and Mauch-Mani, 2004; Ton *et al.*, 2005). *ABSCISIC ACID* (*ABA*)-*INSENSITIVE-8* is allelic to *ELONGATION DEFECTIVE-1* and *KOBITO-1*, which establishes a link between ABA and the cell wall because mutations at this locus disrupt ABA-regulated gene expression, sugar sensitivity, cell elongation, cellulose synthesis, vascular differentiation, and root meristem maintenance (Cheng *et al.*, 2000; Pagant *et al.*, 2002; Brocard-Gifford *et al.*, 2004). ABI8/KOB1/ELD has recently been shown to regulate movement of transcription factors through plasmodesmata to modulate stomatal patterning (Kong *et al.*, 2012). Mutations in the cellulose synthase gene (*CESA8*)/*IRREGULAR XYLEM1* result in resistance against drought, salt, and osmotic stresses, and in elevated ABA-inducible gene expression (Chen *et al.*, 2005).

Because there is no cell migration in plants, and cell walls are formed concomitant with cell division, the timing and orientation of cell division and expansion, mediated ultimately by hormone signalling to the cell wall, are the primary forces that shape plants. Identification and cloning of mutants that affect plant shape and environmental responses can shed light on the molecular mechanisms that control morphogenesis and hormone action in response to stresses. So far, it has proved difficult to predict the nature of the gene products involved in hormone sensitivity. Previously the *harlequin* (*hlq*) mutant was isolated based on ectopic expression of the *proDc3:GUS* transgene (Subramanian *et al.*, 2002). Here it is reported that *hlq*, which is de-etiolated in the dark with open cotyledons and short hypocotyls, encodes an allele of *brassinosteroid insensitive3/hypocotyl6/root hairless3* (*bin3/hyp6/rhl3*), the plant-specific homologue of type IIb archaebacterial topoisomerase VI subunit B.

Type II topoisomerases have a catalytic cycle producing double-stranded breaks (as distinct from type I single-stranded breaks) that are associated with the occurrence of transient DNA–topoisomerase covalent complexes implicated in DNA replication, recombination, chromosome segregation, chromatin remodelling, and transcription (Varga-Weisz *et al.*, 1997; Vos *et al.*, 2011). Type II topoisomerases can act as transcriptional repressors, by binding to promoters and blocking the formation of stable pre-initiation complexes, which can be relieved by the addition of sequence-specific transcriptional activators (Brou *et al.*, 1993; McNamara *et al.*, 2008). Topo6B has conserved ATP binding and hydrolysis domains, and a conserved basic motif (B4) of unknown function (Hartung and Puchta, 2001). In addition to the archaeal TOP6A/BIN5/RHL2/SPO11-3 and TOP6B subunits, *Arabidopsis* TopoVI function requires the activity (Simkova *et al.*, 2012) of two small subunits, *At1g48380* /*RHL1* (Schneider *et al.*, 1998)*/HYP7* (Sugimoto-Shirasu *et al.*, 2005) and *At5g24630/BIN4* (Breuer *et al.*, 2007)/*MIDGET* (*MID*) (Kirik *et al.*, 2007). The *Arabidopsis* BIN4/MID subunit of TopoVI can interact in a yeast two-hybrid assay with TFIIB, which is involved in RNA polymerase II recruitment and transcription initiation in eukaryotes (Evans-Roberts *et al.*, 2010; Szklarczyk *et al.*, 2011). Molecular characterization of mutants of *bin3/hyp6/rhl3/top6b* and the regulatory subunits *rhl1/hyp7* and *bin4/mid* that bind each other, to TOP6A, and to COP1 (Schrader *et al.*, 2013) led to claims of TopoVI as having complex roles in growth and development. Namely, it is essentially involved in brassinosteroid sensitivity (Yin *et al.*, 2002) but probably not hormone cross-talk (Nemhauser *et al.*, 2006), in decatenation of endoreduplicated chromosomes throughout the nucleus (Sugimoto-Shirasu *et al.*, 2002), in DNA replication/repair and coupling to cell cycle arrest that controls cell expansion and proliferation (Hartung *et al.*, 2002; Schrader *et al.*, 2013), including in response to nematode parasites (Vieira *et al.*, 2013). Moreover, TopoVI is involved in transcriptional silencing through chromatin organization (Kirik *et al.*, 2007), and as a genuine component of singlet oxygen retrograde signalling to the nucleus, being both a positive and a negative integrator of different reactive oxygen species (ROS) signals and environmental cues (Simkova *et al.*, 2012). Further studies have shown that the constitutive expression of rice OsTOP6A or OsTOP6B increases the expression of stress-responsive genes, and confers abiotic stress tolerance to transgenic *Arabidopsis* plants (Jain *et al*., 2006, 2008). The present phenotypic characterization and transcriptome profiling results for *hlq* seedlings now demonstrate a fundamental role for TopoVI in plant gene regulation, and provide additional insight into the molecular mechanisms and processes of TopoVI which integrate environmental (e.g. light and dark) and internal (e.g. hormone) signals via chromatin remodelling to control plant growth and development.

## Materials and methods

### Plant materials

Col-0 (stock # CS60000), *abi2-1* (CS23), Ler (CS20), *prc1-1* (CS297), *bot1-1* (Bichet *et al.*, 2001), *kor1-1* (CS298), and T-DNA insertion lines SALK_024455C (subsequently referred to as the *hlq-3* allele) and SALK_140704 (*hlq-2*) were obtained from the Arabidopsis Biological Resource Center (Ohio State University, Columbus OH, USA; http://abrc.osu.edu/). The *hlq-1*/+ (CS68702), F_2_ mapping stocks (CS68703, CS68704), *hlq-2*/+ (CS68705), and *hlq-3*/+ (CS68706) seed stocks have been deposited at the ABRC. Seeds were sown on plates containing 0.5× Murashige and Skoog salts (Research Products International, Mt. Prospect, IL, USA), 0.8% sucrose, and 0.5% phytagel (Sigma-Aldrich, St. Louis, MO, USA). Plates were kept in the dark at 4 °C for 3 d before being transferred into a growth chamber. The growth conditions were 21 °C with continuous light (~ 100 μE m^–2^ s^–1^), except for starch quantitation assays where plants were given a diurnal light cycle of 12h light and 12h dark, and roots and shoots (four biological replicates of ~10mg each) were harvested at the end of the respective cycle. Plates were kept in the chamber for 5 d before scoring for the *hlq* mutant morphology.

### Genetic mapping

Previously, 5100 M_1_ *abi2/abi2* homozygous plants of a line that carried two independent *proDc3*:*GUS* reporter genes were mutagenized with ethylmethane sulphonate (EMS), and M_2_ clonal lines were screened for ABA- or auxin-inducible GUS expression in roots. These studies resulted in isolation of a new allele of *rooty/superroot1/hookless3* (Sun, 2003) and two single gene nuclear mutants: *hlq* and *short blue root* (*sbr*) (Subramanian *et al.*, 2002). These mutants have novel ABA- and auxin-inducible *proDc3:GUS* gene expression phenotypes attributable to ABA and indole acetic acid (IAA) responses, as well as pleiotropic growth phenotypes that suggest a link to ABA and/or auxins. The *hlq*/+ mutant stock (subsequently termed *hlq-1* allele) were backcrossed to the parental line *abi2* three times to remove extraneous mutational load caused by the original EMS treatment and to provide supporting evidence that the pleiotropic phenotypes of *hlq* were due to a single mutation. Due to seedling lethality and sterility, *hlq*/+ heterozygous plants (in the *abi2-1* mutant background) of the Landsberg *erecta* (Ler) ecotype were crossed with the Columbia (Col-0) ecotype. F_2_ seeds from a single F_1_ plant constituted one mapping population. Two F_2_ lines that segregated for *hlq* were used for mapping experiments. The segregation in the F_2_ population for 3:1 wild type to *hlq* showed that the *hlq* phenotype was not influenced by the ecotype. No obvious differences in the *hlq* mutant morphology compared with the Ler parental background suggested that penetrance of the phenotype was complete. DNA was extracted from 2- to 3-week-old mutant seedlings as described (Xin and Chen, 2012). The *hlq* recombinants were allowed to grow for 3–4 weeks to maximize template DNA yield. The DNA from individual recombinants was used in simple sequence length polymorphism (SSLP) (Bell and Ecker, 1994) and cleaved amplified polymorphic sequence (CAPS) PCR analyses (Konieczny and Ausubel, 1993), or amplified using primers flanking single nucleotide polymorphisms (SNPs) identified from the publicly available Monsanto/Cereon Arabidopsis Ler draft sequence (Jander *et al.*, 2002) [available for download at The Arabidopsis Information Resource (TAIR) (www.arabidopsis.org)]. Repetitive sequences were scanned using the REPEATMASKER program (www.repeatmasker.org). Oligonucleotide primers were designed using ‘Perlprimer’ (http://perlprimer.sourceforge.net/) and synthesized commercially (Sigma; http://sigma.com) and used for PCR amplification using PrimeSTAR HS DNA Polymerase (TAKARA, Shiga, Japan). Amplicons of 100–200bp were targeted to reveal the longest repeats and hence the high probability of polymorphism in agarose gels. Among the different kinds of repeats scanned, TA, CT/CTT, and GA/GAA repeats showed highest abundances and successes for developing useful polymorphic markers at 24, 22, and 14%, respectively, of the total scanned. Information about the position and primer pairs for these markers is given in Supplementary Table S6 available at *JXB* online. Amplicons were desalted with a Qiaquick PCR Purification Kit (Qiagen, Gaithersburg, MD, USA) and sequenced by the Sanger dideoxynucleotide chain termination method by Beckman Coulter Genomics (Danvers, MA, USA; http://www.beckmangenomics.com/).

### Whole-genome resequencing of the *hlq/+* heterozygote

Genomic DNA was prepared with a Qiagen DNeasy Plant Minikit (Qiagen) according to the manufacturer’s protocol. Paired-end 100 base Illumina HighSeq reads as fastq files (including quality scores) were mapped to the Col-0 reference genome (TAIR9) using Burrows–Wheeler Aligner (BWA; http://bio-bwa.sourceforge.net/) software with default settings, except the extension gap was set to ‘10’ (Li and Durbin, 2010). This resulted in a slight improvement to 45.7% of all reads successfully mapping to the reference Col-0 genome, and 38.7% of all reads properly paired, yielding an average of 25-fold coverage over all five chromosomes. SAMTools (http://samtools.sourceforge.net/) (Li *et al.*, 2009) was used to index and align the reads to the reference genome, and SnpEff (http://snpeff.sourceforge.net) was used to call SNPs and INDELs (Cingolani *et al.*, 2012). There were 223 homozygous Ler SNPs called in the ~112 kbp interval of interest where *hlq* mapped in the region chr3: 7213133..7325482, plus 19 deletions and 18 insertions. There were 11 heterozygous Ler SNPs called, one heterozygous insertion, and one heterozygous deletion. A published whole-genome assembly of Ler with >320-fold coverage (Schneeberger *et al.*, 2011) was used to validate the SNP calling methodology. Both heterozygous INDELs were called correctly, as were 92% of the Ler homozygous SNPs/INDELs in the interval (data not shown), whereas an additional 15 SNPs (7%) called by Schneeberger *et al.* (2011) were not called in the present analysis. It is possible that the extra homozygous SNPs in the data set here and those described but not found in the data are polymorphic within Ler accessions. Notwithstanding, the high concordance between the present results and the published Ler SNPs validated the method.

### GUS assays

Plant samples were developed at 37 °C for 12h with a 1mM solution of the indigogenic GUS substrate 5-bromo-4-chloro-3-indolyl β-d-glucuronide (X-Gluc; Rose Scientific, Edmonton, Alberta, Canada) in 50mM KH_2_PO_4_ (pH 7.0), 0.1mM EDTA, 0.5mM ferricyanide, 0.5mM ferrocyanide, 0.05% sodium azide, 0.1% Triton X-100. After staining overnight, samples were immersed in 70% ethanol overnight. Samples were then mounted in 30% glycerol on microscope slides for pictures to be taken with a dissecting microscope, or were imaged with an Olympus BX41 light microscope for high magnification detailed imaging using QCapture software (v2.68.6, Silicon Graphics, Fremont, CA, USA).

### Analysis of gene expression and genotypes with PCR

**Fig. 4.**
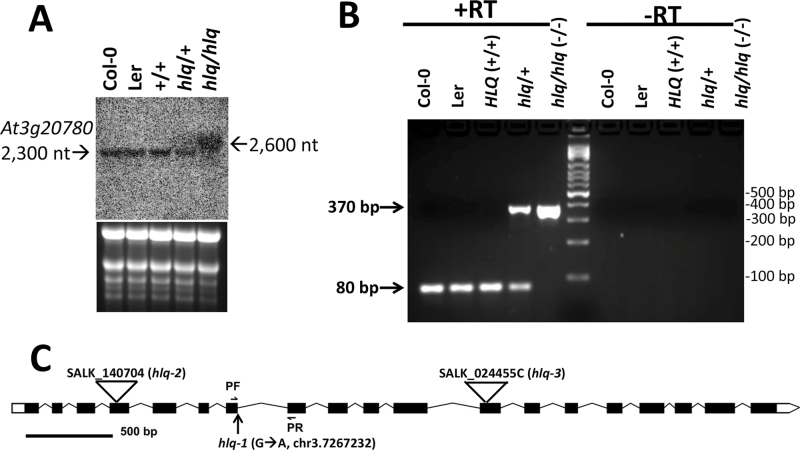
Characterization of the *At3g20780* mRNA in the *hlq* mutant demonstrates aberrant intron 7 splicing. (A) RNA gel blot of *At3g20780* shows an aberrant mRNA in *hlq* homozygotes. A 20 μg aliquot of total RNA from seedlings of wild types Col-0, Ler, and HLQ+/+ (*abi2-1* parental background), *hlq*/+ heterozygotes, and the homozygous *hlq/hlq* mutant were probed with a 1.5 kbp fragment of the *At3g20780* cDNA; the lower panel shows equal loadings by ethidium bromide staining of the denaturing agarose gel before blotting. Band sizes of the wild-type and *hlq At3g20780* mRNAs are labelled on the left and right sides, respectively. (B) Agarose gel of RT–PCR amplicons from cDNA prepared from total RNAs of control (Col-0, Ler, and HLQ/HLQ), *hlq/+* heterozygotes, and *hlq/hlq* homozygotes (–/–) shows retention of intron 7 (355bp predicted) in *hlq* genotypes, and absence of intron 7 (~80bp amplicon, 55bp predicted) in controls. ‘+RT’, with reverse transcriptase; ‘–RT’ reverse transcription step omitted from the amplification protocol. (C) Cartoon showing the exon–intron–untranslated region (UTR) structure of *At3g20780*, the position of primers (PF, PR; horizontal arrows) used for PCR in B, the position of the *hlq-1* mutation (vertical arrow) at the intron 7 splice donor site, and positions of T-DNA insertions (triangles) in lines SALK_024455C (*hlq-3*) and SALK_140704 (*hlq-2*).

Total RNA from roots or leaves of 10-day-old seedlings, or from whole seedlings, was extracted with Iso-RNA Lysis Reagent (5 PRIME, Gaithersburg, MD, USA). For reverse transcription–PCR (RT–PCR), 2 μg of total RNA was digested with RQ1 RNase-free DNase (Promega, Madison, WI, USA) and reverse transcribed by MMLV reverse transcriptase (Promega) with Anchored Oligo-dT (ABgene; Surrey, UK). A cycle number of 30 was used for PCR experiments in Fig. 4B; for Supplementary Fig. S7 at *JXB* online, 28 cycles were used for SALK_140704, and 31 cycles for SALK_024455. PCR conditions were: 55 °C annealing temperature, 1.5min extension time, 72 °C extension temperature. PCR products were run on an agarose gel with a DNA 1 kbp ladder (Axygen Biosciences, Union City, CA, USA; www.corning.com/axygen/) for calculation of band sizes by linear regression. Primers used in this study are listed in Supplementary Table S6.

### RNA gel blot hybridization

For RNA gel blot hybridization, samples of 20 μg of total RNA were resolved on a 1.2% denaturing agarose gel and blotted to a Hybond-N+ membrane (GE Healthcare, Piscataway, NJ, USA) according to the supplier’s protocol. Ambion Millenium RNA markers (GE Healthcare) were run as controls for calculation of band sizes by linear regression. *At3g20780* template was amplified using the primers listed in Supplementary Table S6 at *JXB* online from a cDNA library and was eluted after excision of the band from an agarose gel. Probes were synthesized using the Random Primer DNA Labeling Kit Ver.2 (TAKARA) with [α-^32^P]dCTP (PerkinElmer, Waltham, MA, USA). Hybridization was carried out with the PerfectHyb Plus hybridization buffer (Sigma-Aldrich) according to the manufacturer’s instructions. A storage phosphor screen (GE Healthcare) was used for autoradiography and it was scanned using a Storm 860 PhosphorImager (GE Healthcare). Band intensities were quantified using the ImageQuant TL software (v2003, GE Healthcare).

### Microarray experiment

Three individual pools of total RNA were extracted with Trizol reagent (Invitrogen, Carlsbad, CA, USA) from ~1000 seedlings (14 d old) segregating *hlq* homozygotes from self-fertilization of the *abi2-1/abi2-1-hlq/+* genotype after germination on Petri plates as described and kept vertically under continuous light at 21 °C. The control (wild-type) samples were three pools of normal individuals from the same plates and comprised ‘wild type’ (i.e. *abi2-1/abi2-1* homozygous) and *abi2-1/abi2-1*-*hlq*/+ heterozygous genotypes used for microarray analysis. *In vitro* transcription was carried out using an Amino Allyl MessageAmp aRNA Amplification Kit (Ambion; cat#: AM1753) according to the manufacturer’s protocol (Grand Island, NY, USA). A 1000ng aliquot of Cy3- or Cy5-labelled cRNA from different replicate genotype samples was purified separately, combined in equimolar amounts including dye swaps across genotypes, and mixed with hybridization buffer before being applied to three microarrays. Each sample was run on a Bioanalyzer (Agilent Technologies, Santa Clara, CA, USA) before and after labelling. The ratio of red (Cy5) and green (Cy3) fluorescence intensities for each spot is indicative of the relative abundance of the corresponding target molecule in the two mRNA target samples. A fourth microarray was hybridized with balanced pooled samples from the same dye-labelled biological replicates as a technical replicate. Hybridizations were performed on the Operon Arabidopsis Long Oligonucleotide Microarrays V3 GEO platform GPL4570. Conditions of hybridization and washing were according to the protocol published by Galbraith *et al.* (2011). After washing, microarrays were scanned using the GenePix Autoloader 4200AL Scanner (Molecular Devices, Sunnyvale, CA, USA) with laser excitation at 532nm and 635nm at the resolution of 10 pixels μm^–1^, and saved as 16-bit greyscale multi-image TIFF files. Intensity values were extracted using GenePix Pro 6.0 (Molecular Devices) and saved as ‘gpr’ and ‘txt’ files for each block individually. The data for each array were lowess normalized followed by analysis of variance (ANOVA) of differential gene expression using empirical Bayes methods to moderate the standard deviations between genes. This was done by the software package Linear Models for Microarray Data (limma) (Smyth *et al.*, 2005) written in R language under the BioConductor platform (Gentleman *et al.*, 2004) version R2.5.1 (http://www.r-project.org/). Normalization ‘within’ and ‘between arrays’ scaled the log2-ratios of each two-colour experiment to have the same median-absolute deviation across arrays. A dye effect coefficient was calculated in addition to the genotype coefficient and signal amplitude across all probes and samples. Original MIAME-compliant data are stored at the Gene Expression Omnibus (http://www.ncbi.nlm.nih.gov/geo/) with the locator GSE45806. The key reporters referenced in the literature for retrograde ROS signalling [with fold change (FC) observed in *hlq* seedlings following in parentheses] are: *At5g40010/AAA-ATPase1* (FC=1.08), *At3g61190/BONSAI ASSOCIATION PROTEIN1* (FC= 1.68, *P*<0.0005), and *At5g01600/FERRITIN1* (FC= 1.48, *P*<0.03). The lack of significant up-regulation for *AAA-ATPase1* in *hlq* mutants is consistent with previous results for 5-day-old seedlings of *top6a/caa39* (Simkova *et al.*, 2012).

### Lignin, callose, pectin, and starch staining/quantitation, dichlobenil and tunicamycin treatments

For staining for lignin with phloroglucinol, the seedlings were treated with a mixture of methanol:acetic acid (9:1) to remove chlorophyll. The seedlings were then cleared in chloral hydrate:water (8:2) for 2–5min and stained with phloroglucinol (1% phloroglucinol in 6 N HCl) for 5min (Zhong *et al.*, 2000). Stained seedlings were observed and photographed using an Axio-vert inverted microscope or Axiophot microscope. Inverse phase contrast was used to better visualize lignin staining. For staining lignin with basic fuchsin, the seedlings were treated with a mixture of methanol:acetic acid (9:1) to remove chlorophyll and then cleared in 10% NaOH at 60 °C for 12h. Cleared seedlings were stained with 0.01% Basic Fuchsin, mounted in 50% glycerol, observed by epifluorescence using a propidium iodide (PI) filter, and photographed.

Callose staining was performed with slight modifications from Buogourd *et al*. (2000). The seedlings were treated with a mixture of methanol:acetic acid (9:1) to remove chlorophyll and then re-hydrated in water. The seedlings were stained with a 0.025% aqueous solution (1 in 20 dilution of 0.5% stock solution) of aniline blue (Sigma-Aldrich) for 30min. The seedlings were then washed twice with water for 15min each to remove excess aniline blue, mounted in 50% glycerol, observed by epifluorescence, and photographed using a 510nm filter. Callose was quantified from seedlings of different ages according to Kohler *et al.* (2000). Briefly, the seedlings were weighed, ethanol dehydrated and decolorized for 2 d, homogenized, and extracted with the loading mixture (0.1% aniline blue, 1M glycine/NaOH, 1M HCl). The fluorescence was measured in a fluorescence spectrophotometer at 393nm excitation and 479nm emission wavelengths.

Starch staining was with Lugol’s elemental iodine:potassium iodide (IKI; 5.7mM iodine, 43.4mM potassium iodide) after pigment removal and dissolution of membranes by boiling in 80% ethanol, as described (Kurata and Yamamoto, 1998). Starch quantitation was by solubilization (boiling pulverized frozen material in water after extracting free glucose with 90% ethanol, 60 ºC for 5min), complete conversion with glucan hydrolases to glucose, followed by enzyme-based fluorimetric assay (Abcam; www.abcam.com) according to the manufacturer’s protocol.

For staining pectins, the seedlings were stained with Ruthenium Red (SPI-Chem, Cat. No. 02603-AB) at 0.2% in water with 0.01% Tween for 1h, rinsed twice in distilled water, and viewed under a dissection microscope. Tunicamycin or 2, 6-dichlorobenzonitrile (dichlobenil, DCB; Sigma-Aldrich) was added to the growth medium at a concentration of 1, 2, or 3 μM. Two-day-old seedlings were transferred to tunicamycin-containing medium and allowed for grow for a further 5 d. Their growth was monitored and phenotypes observed on 1-week-old seedlings.

### Peroxidase activity

Four-week-old wild-type and *hlq* mutant plants grown on agar plates were homogenized at 4 °C in homogenization medium [10× vol. g fresh weight (FW)^–1^] comprising 50mM TRIS-HCl (pH 7.0), 2 μM phenylmethylsulphonyl fluoride, and 1 μg ml^–1^ of each of the protease inhibitors leupeptin, pepstatin A, and aprotinin (Sigma). The homogenate was centrifuged at 3000 *g* for 15min to obtain a soluble and crude cell wall fraction. The pellet was washed in homogenization medium and then incubated with 1M KCl (2× vol. g FW^–1^) in 50mM TRIS-HCl buffer (pH 7.0) for 10min prior to centrifugation for 10min at 3000 *g* to obtain the KCl extract. The KCl extract was microfuged at 13 000rpm for 7min at 4 °C to remove cell debris and concentrated 10-fold by ultrafitration using a 1.5ml Fugisep (10kDa molecular weight cut-off membrane; Intersep). The KCl extracts were equilibrated in 20mM TRIS-HCl (pH 7.0) by diafiltration using Fugisep-10 membranes (Intersep) prior to loading on a 10% native-polyacrylamide gel (Brownleader *et al.*, 2000). Electrophoresis was performed at 4 °C at 30 mA constant current. The gel was then immersed in a solution of 0.1% guaiacol and 0.03% H_2_O_2_ in 50mM potassium acetate buffer, pH 6.0 until bright blue bands appeared (7–10min). The reaction was stopped by immersing in 7% acetic acid.

### Microscopy

Light microscopy was performed using a Zeiss stereomicroscope (Göttingen, Germany), and for micro measurements a calibrated microscale was used. For light microscopy of GUS-stained roots, the seedlings were briefly rinsed in 70% ethanol followed by sterile distilled water to remove excessive GUS developer and observed under the microscope. For ‘live and dead’ cell staining, seedling roots were embedded by allowing them to grow into 2% Phytagel minimal medium. Embedded roots in 1–2mm thick slices of gel were transferred to glass slides, immersed in water containing 100 μg ml^–1^ each of fluorescein diacetate (FDA; Sigma) and PI (Sigma), then incubated in the dark (15min), rinsed twice in sterile distilled water, and epifluorescence was viewed under excitation with a blue filter (450–490nm) by means of a Zeiss Axiophot microscope. The sample was then flooded with GUS developer solution for 16h and photographed in bright field with the same field in view as previously documented. For aniline blue visualization, excitation was with UV and emission was visualized with a 4’,6-diamidino-2-phenylindole (DAPI) filter set.

For scanning electron microscopy, the seedlings were aligned on the conductive paste thinly spread over the microscope stage and immediately frozen under liquid nitrogen. The stage was placed into the cryo-chamber of the microscope and the condensed water removed by vacuum pumping. Then the samples were coated with platinum and observed under a Leica S440 scanning electron microscope (Cambridge, UK) fitted with a Fisons LT7480 cryoprep cryo-stage (Fisons Instruments, UK).

### Fourier transform infrared (FTIR) microspectroscopy

FTIR has been used to ‘fingerprint’ the carbohydrate constituents in the 1200–900cm^–1^ region, to detect and characterize conformational changes in wall components, and to determine which cross-links between polymers are present. Diagnostic absorbances are as follows: the carboxylic ester group absorbs at ~1740cm^–1^, amide-stretching bands of protein occur at ~1650cm^–1^ and 1550cm^–1^, carboxylic acid groups on pectins absorb at 1610cm^–1^, phenolics absorb at ~1600cm^–1^ and 1500cm^–1^, and carbohydrates absorb between 1200cm^–1^ and 900cm^–1^ (McCann *et al.*, 1992). IR spectra of *hlq* and the wild type were acquired with the Autoimage FT-IR micro-spectroscopy system (PerkinElmer; Norwalk, CT, USA). The microscope includes a camera and a viewing system that magnifies the visible light image of the sample and enables isolation of a region of interest. The image of the sample is displayed on the monitor visible window. The AutoIMAGE software (version 5.0.0 B8) enables the control of the operation of the microscope, and maps and collects the spectra from a sample. The system used a KBr beam splitter and utilized a liquid nitrogen-cooled MCT detector housed in the microscope. The Autoimage was further equipped with an automated XYZ motorized stage that was operated in the auto-focusing (Z-direction) mode under the software control. Both visible images and IR maps were obtained in the transmission mode from an area of 150×150 μm. Samples of cell wall extracts (described below) were placed on a BaF_2_ window (13mm diameter, 2mm thickness) and air dried at 37 °C for 1h. A background spectrum of clean BaF_2_ surface was collected before each scan. The IR spectra were recorded using 128 interferograms with 4cm^–1^ spectral resolution between 4000cm^–1^ and 700cm^–1^. Twelve spectra were recorded from each sample. All FTIR spectra were baseline corrected and normalized. Principal component analysis (PCA) is a mathematical technique that is widely used to reduce the dimensionality of data sets. The variability in each individual spectrum relative to the mean of the population is represented as a smaller set of values (axes) termed principal components (PCs). This process concentrates the sources of variability in the data into the first few PCs, and was used in analysis of FTIR spectra from purified cell walls of *hlq* homo- and heterozygotes, and the wild type.

### Glycosyl composition analysis of cell walls

Plant tissue (0.7–2g FW) was collected and stored at –70 °C until processed. Frozen tissue was ground into fine powder in liquid N_2_ with a mortar and pestle and extracted twice in 80% (w/w) phenol:acetic acid:water (5:2:1 v/v/v) to remove protein and other soluble metabolites (Fry, 2000). The pellet after centrifugation was washed with 70% ethanol and extracted twice with 90% (v/v) dimethylsulphoxide (DMSO) to remove starch (Selvendran and O’Neill, 1987). Lipids were removed by washing the pellet twice with chloroform:methanol (2:1 v/v) and acetone (York *et al.*, 1986). The cell wall fraction was dried in air and again in a vacuum desiccator over P_2_O_5_. The dry cell wall fraction from *hlq* mutant and wild-type plants was then analysed at the level of the glycosyl composition of non-cellulosic polysaccharides as previously described (Komalavilas *et al.*, 1991). Briefly, cell walls were subjected to methanolysis in 1.5M methanolic HCl at 80 °C, trimethylsilylation, and gas–liquid chromatography (GC). For analysis including cellulosic polysaccharides (which are not cleaved by methanolysis), an aliquot of the cell wall fraction was pre-swollen in 22 N H_2_SO_4_, diluted to 1 N H_2_SO_4_, and then hydrolysed at 121 °C (Fry, 2000). After neutralization with Ba(OH)_2_ and removal of BaSO_4_, samples were subjected to methanolysis and trimethylsilylation followed by GC-flame ionization detection (FID) on a Hewlett Packard 5890.

### Bioinformatics

MAPMAN and PAGEMAN software (Usadel *et al.*, 2005, 2006) (http://mapman.gabipd.org) was used for statistical analysis and graphical representation of metabolic and signalling pathways. Dynamic analysis of the results described herein can be recapitulated by installing MAPMAN on a local computer and uploading the *hlq* data set in Supplementary Datafile 1 (sheet 1) at *JXB* online.

## Results

### The *harlequin* mutant manifests pleiotropic phenotypes affecting ABA- and auxin-inducible reporter gene expression, cell elongation, epidermal morphogenesis, and carbon partitioning into primary (starch, cell wall) and secondary metabolites (anthocyanins, lignin)

**Fig. 1.**
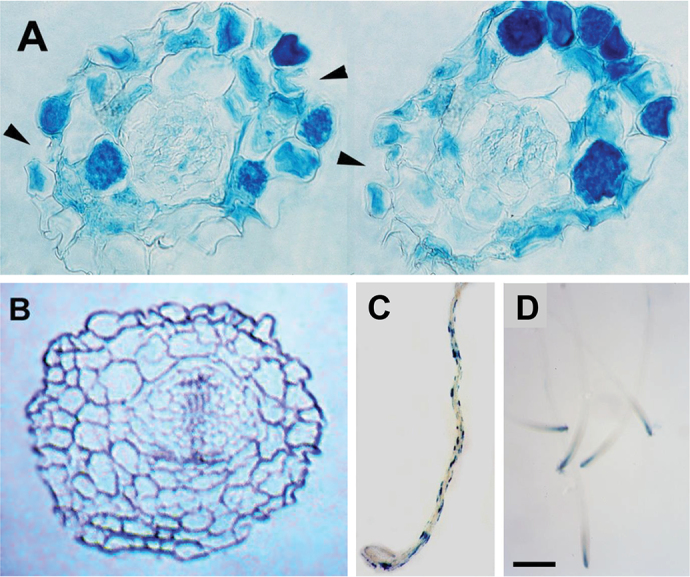
The *hlq* mutant has ectopic expression of the *proDc3:GUS* reporter gene. (A) Cross-section through the hypocotyl of 2-week-old *hlq* and parental-type (B) plants, stained for GUS. Parental-type plants do not stain for GUS after 10 d. *hlq* shows ectopic staining not only in the epidermal cells but also in the cortex. The arrows indicate collapsed cells in the epidermal cell file, and the cell shapes are also irregular. (C) Ectopic expression of *proDc3:GUS* in a 7-day-old *hlq* mutant primary root. (D) Wild-type expression of *proDc3:GUS*. Scale bar=500 μm.

The *hlq* mutant has an extreme dwarf phenotype, with pleiotropic effects on root and shoot morphology, resulting in brittle leaves and stems and more friable callus (Balasubramanian, 2003), radial root tip swelling, anisotropic epidermal cell expansion, root hair defects, and abnormal cell collapse and associated callose accumulation (Supplementary Fig. S1 at *JXB* online) (Subramanian *et al.*, 2002). The shape of the cells in the root cortex and also the epidermis were irregular, unlike the regularly shaped spherical cells in the wild-type tissues (Fig. 1). The *hlq* hypocotyl and root epidermal cell lengths were drastically reduced and the columnar cell files were disrupted (Supplementary Table S1; Fig. S1H), suggesting that the dwarf phenotype of *hlq* is due to a reduction in cell size, and not in cell number, consistent with the results of others indicating that TopoVI is primarily involved in cell expansion by endoreduplication (Breuer *et al.*, 2007; Sugimoto-Shirasu *et al.*, 2005). The leaf pavement cells were less articulated than the degree of interdigitation seen in the parental type, with the presence of gaps between the pavement cells similar to the gaps found in the epidermis of *hlq* hypocotyls and roots (Subramanian *et al.*, 2002). Flower organs of *hlq* plants showed a rough epidermis compared with the smooth surface of the parental-type petals (Supplementary Fig. S1F). These phenotypes are consistent with a role for the *HLQ* gene product in a developmentally modulated pathway that impinges on ABA, light, and cell wall signalling, similar to *ABI8/KOB/ELD1*. *ProDc3:GUS* expression was not specifically confined to either the root trichoblasts or atrichoblasts, as is seen for the *WER* or *GL2* genes (Lee and Schiefelbein, 1999). GUS staining of median cross-sections of the root and hypocotyl of 2-week-old *hlq* plants indicated that the ectopic *proDc3:GUS* expression also extended into the cortex (Fig. 1A).

The *hlq* mutant is chlorotic, with only 50% of the chlorophyll content of the parental type when grown in light (Subramanian *et al.*, 2002), suggesting that cellular metabolism may be broadly affected. When *hlq* pollen was germinated on artificial media, the pollen tubes grew at rates comparable with that of the wild type (data not shown). However, out- or back-crosses of *hlq* did not yield seeds, nor did manual pollination of *hlq* mutant flowers with the parental-type pollen. The *hlq* mutant plants failed to survive on soil or on basal minimal agar media, but under conditions of 0.8% sucrose supplementation, *hlq* homozygous plants could survive to the flowering stage, although vegetative tissue lost chlorophyll and leaves appeared brown/red in colour (data not shown).

The ability of *hlq* mutants to survive to flowering when grown on sucrose prompted investigation of carbon partitioning into starch, since it serves as a short-term carbohydrate reservoir. The *hlq* mutant in the Ler genetic background appeared to accumulate anthocyanins in the shoot meristematic region (Supplementary Fig. S1C at *JXB* online), reminiscent of the *de-etiolated/constitutive photomorphogenesis/fusca* class of mutants and transgenic *Arabidopsis* overexpressing ABI3, ABI4, and ABI5 transcription factors (Finkelstein *et al.*, 2002), but not when in the *abi2-1* parental background (data not shown). This is possibly because the *abi2* mutation has been shown to suppress sugar induction of the transcription factors *PRODUCTION OF ANTHOCYANIN PIGMENT1*(*PAP1*)*/MYB75* and *PAP2/MYB90* ~6- and 9-fold, respectively, relative to wild-type Ler (Luo *et al.*, 2012).

**Fig. 2.**
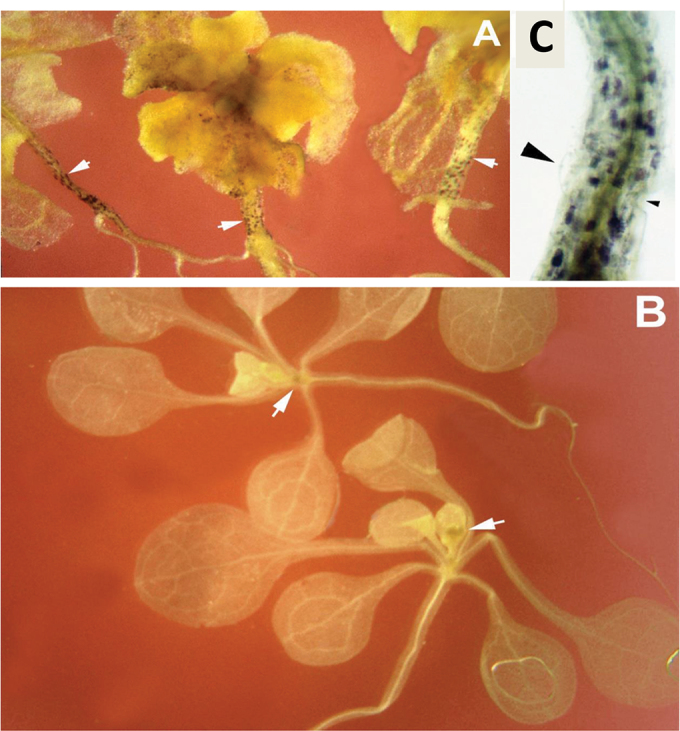
Ectopic localization of starch in *hlq* mutants. Staining for starch granules by KI shows ectopic localization of starch in the hypocotyl and leaves of *hlq* (A, white arrows), whereas there is no staining in the wild-type except at the meristem (B, white arrows). A close-up view of *hlq* hypocotyl (C) shows that the starch localization does not strictly correspond to *hlq* specific cell types—neither a bulged, radially expanded cell (large black arrowhead) nor a collapsed cell (small black arrowhead).

Sugar partitioning into starch was assessed by staining *hlq* mutants with IKI (Kurata and Yamamoto, 1998) after 10h of darkness. The *hlq* mutants showed patchy staining of starch granules in the hypocotyls and in the leaves (Fig. 2A), while the wild type was completely devoid of starch (Fig. 2B). The ectopic deposition and relative abundance of starch in shoots of *hlq* mutants was reminiscent of the *proDc3:GUS* staining pattern. However, counterstaining the GUS-stained *hlq* seedlings with IKI showed no obvious concordance between cells expressing GUS and those accumulating starch. Furthermore, the bulged and collapsed hypocotyls cells did not correlate with the presence of starch (Fig. 2C). Together, these results suggested that *hlq* has either decreased starch breakdown or increased starch synthesis rates, or defects in carbon metabolism or partitioning, which might also affect flux into secondary metabolites such as phenolics (e.g. anthocyanins and lignins). Starch was subsequently quantified in *hlq* and two new alleles (see below).

Caño-Delgado *et al*. (2000) showed that cell elongation defects lead to ectopic lignification. Their results were independently confirmed here with the *korrigan1* mutant (data not shown) and ectopic lignin was tested for within the *proscute1-1/AtCES6* cellulose-deficient mutant (Fagard *et al.*, 2000) and *botero1-1*, a mutant allele of a katanin-like protein thought to be involved in microtubule stability (Bichet *et al.*, 2001). It was found that both mutants displayed ectopic lignification within the root (Supplementary Fig. S2D, E at *JXB* online). In order to investigate the relationship between the cell wall and hormone- and stress-regulated gene expression, the expression of the *proDc3:GUS* reporter was examined in the *prc1-1* and *bot1-1* cell wall mutants. The *proDc3:GUS* marker gene was crossed into the *prc1-1* and *bot1-1* mutant backgrounds using a *Ler* double insertion line (P series) (Chak *et al.*, 2000) as one parent. In the F_2_ progeny, the expression patterns of *proDc3:GUS* in mutant individuals were visualized with the chromogenic substrate X-Gluc. Roots of *prc1-1* had varying degrees of constitutive GUS expression (Supplementary Fig. S2B), but were clearly distinguishable from wild-type plants (Supplementary Fig. S2A) on the basis of their *proDc3:GUS* staining intensity. Individuals of *bot1-1* also displayed a range of *proDc3:GUS* expression phenotypes (Supplementary Fig. S2C). The extent of *proDc3:GUS* expression correlated with the extent of severity of the dwarf phenotypes in the *bot1-1* population (based on root length). These results taken together suggest that cell wall mutants phenocopy the ectopic expression of *proDc3:GUS* seen in *hlq* and raise the question of whether the *hlq* mutant also manifests ectopic lignification.

Significantly, 2 weeks after germination, *hlq* mutant plants showed conspicuous swelling of the root elongation zone (Supplementary Fig. S1D at *JXB* online), whereas swelling was less obvious earlier in development (data not shown). The swelling appeared similar to the phenotype of cellulose deficiency for *radially swollen1*, which encodes a glycosyl transferase (Arioli *et al.*, 1998), and the phenotype of the *cytokinesis defective1* mutant, which encodes a GDP-mannose pyrophosphorylase required for *N*-glycosylation (Lukowitz *et al.*, 2001). Treatment of wild-type seedlings with the herbicide dichlobenil (DCB; 2,6-dichlorobenzonitrile), an inhibitor of cellulose synthesis, phenocopied the swelling observed in the *hlq* root elongation zone. In the presence of 1 μM DCB, wild-type plants developed a swollen root differentiation zone with few root hairs (Supplementary Fig. S3F). A similar effect was induced by treatment with 1 μM tunicamycin, which interferes with the synthesis of the core glycan chain attached to the Asn-Xaa-Ser/Thr motif of *N*-linked glycoproteins, which resulted in severe dwarfing, abnormal swelling of the root, and epidermal cell bulging (Supplementary Fig. S3A), as described by Lukowitz *et al.* (2001). Root hairs emerged closer to the root tip, suggesting inhibition of cell elongation that led to a shorter root elongation zone. The hypocotyls of wild-type plants grown on tunicamycin were also short and radially swollen, with abnormally bulged epidermal cells (Supplementary Fig. S3B) similar to those observed (Fig. 2C) for untreated *hlq* seedlings. Furthermore, when stained for *proDc3:GUS* activity, the tunicamycin-treated wild-type plants exhibited ectopic GUS expression (data not shown), consistent with results from cell wall mutants.

**Fig. 3.**
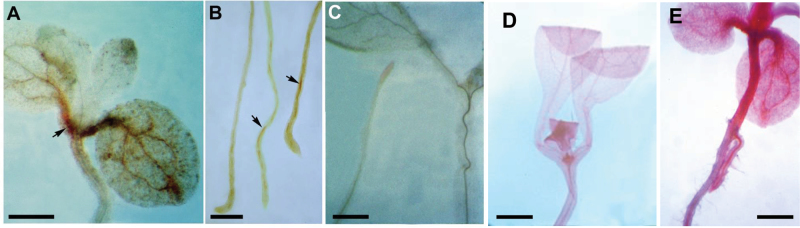
Ectopic expression of lignin and pectin in *hlq* seedlings. Phloroglucinol staining for lignin in *hlq* shoot (A) and roots (B) shows ectopic deposition of patches of lignin (arrows). Wild-type (C) staining for lignin showed a faint signal only in the vasculature. Bars=1mm for (A, C); 2mm for (B). (D, E) Ruthenium Red staining for pectin. Ten-day-old wild-type seedlings (D) showed faint staining in the vasculature, meristem, and leaf primordium, whereas there was ectopic deposition of pectin in *hlq* (E) throughout the vasculature, leaves, and hypocotyl. Bar=1mm.

Similar to the phenocopy of *hlq* for root swelling by inhibitors of cellulose biosynthesis, root tips of tunicamycin-treated seedlings had ectopic lignification, whereas root differentiated regions were normal (Supplementary Fig. S3C at *JXB* online; compare differentiated root region on the left with root tip on the right). Staining for lignin with phloroglucinol showed ectopic staining in the cotyledons and roots of *hlq* (Fig. 3A, B), while the wild type showed only a faint staining in the vasculature (Fig. 3C). Histochemical staining of *hlq* mutants for the presence of suberin using Sudan Red did not show differences from the wild type. Finally, staining with Ruthenium Red, which specifically identifies pectins (Shevell *et al.*, 1994), indicated ectopic pectin accumulation throughout the hypocotyl and cotyledons of *hlq* mutants (Fig. 3E), which compares with the detection of pectin only in the vasculature, meristem, and leaf primordia of wild-type plants (Fig. 3D). Taken together, these results suggested that the *hlq* mutant has defects in carbon partitioning to starch and in cell wall biosynthesis impacting primary (cellulose, pectin) and secondary carbon metabolism, for example lignin and anthocyanins.

### *hlq* is an allele of *BRASSINOSTEROID INSENSITIVE3*/*ROOT HAIRLESS3/HYPOCOTYL6/TOPOISOMERASE6 SUBUNIT B*

The *hlq* mutant was previously mapped to the short arm of chromosome 3, ~6.6 cM south of the SSLP marker *nga162* (Subramanian *et al.*, 2002). The locus was further fine-mapped by analysing another 3832 F_2_ recombinant chromosomes segregating as homozygous mutants from two self-pollinated F_1_ plants (976 and 940 recombinant progeny, respectively) obtained from a cross between *hlq/+* heterozygotes and the ecotype Col-0. The results placed *hlq* in an ~112 kbp interval within bacterial artificial chromosome (BAC) clones F3H11 (>coordinate chr3:7213133), MOE17, and MDF22 (<coordinate chr3:7325482), a region that contains 30 annotated genes. The first indications as to the possible molecular defect in *hlq* came from the observation that another mutant, *hyp6*, characterized by K. Sugimoto-Shirasu (personal communication, XIIIth International Conference on Arabidopsis Research) mapped near to *hlq* and shared similar phenotypes of anisotropic growth, radial swelling of epidermal cells, strongly reduced hypocotyl elongation, immature trichome development, suppressed root hair formation, and enriched cell wall pectins (Balasubramanian, 2003).

A candidate G→A mutation (TAIR10 coordinate chr3: 7267232) that abolished the intron 7 donor consensus (nucleotide 1 of 300) in the pre-mRNA of *At3g20780*/*BIN3/HYP6/RHL3/TOP6B* was identified by Illumina HiSeq 2000 whole-genome DNA resequencing of *hlq/+* heterozygotes. Supplementary Fig. S4 at *JXB* online shows the results of Sanger sequencing with a double peak at the mutation site in amplicons from *hlq*/+ heterozygous template genomic DNA, confirming the Illumina short read analysis. The predicted effect of the mutation, assuming no splicing of intron 7 followed by translation of the aberrant 2512 nucleotide mRNA (based on TAIR10 annotation), is a truncated protein lacking 472 C-terminal amino acid residues of the normal full-length 670 amino acid protein, with the peptide IIIYSYQV added to Met198 before a nonsense UGA stop codon at nucleotide 25 of intron 7. The functional consequence of the translated mutant mRNA would be mutation of a highly conserved aliphatic residue, two residues removed from the GXG motif of the ATP-binding site in the ATPase-like domain (amino acids 49–215; pfam accession PF02518; http://www.ncbi.nlm.nih.gov/Structure/cdd/cddsrv.cgi?uid=238030) and loss of the DNA TopoVI subunit 6B transducer domain (amino acids 398–557; pfam accession PF09239) (Corbett and Berger, 2006).

In order to provide supporting evidence for the G→A transition mutation being causal for the *hlq* phenotypes, the effect of the mutation was characterized at the mRNA level. Figure 4 shows the results of a total RNA gel blot from seedlings segregating *hlq*/+ heterozygotes (2:1 ratio with wild-type plants) and homozygous *hlq* mutant plants, probed with a 1.5 kbp amplicon encompassing most of the *At3g20780* mRNA (2212 nucleotides). There was a reduction in the relative abundance of the wild-type *At3g20780* mRNA (~2300 nucleotides calculated by linear regression of the mobilities of the RNA size markers) in heterozygotes, and a complete absence of the wild-type mRNA species in *hlq* homozygous plants. This was replaced by a diffuse band, ~300 nucleotides larger (Fig. 1A), which is consistent with the prediction that the mutation disrupts splicing of the 300 nucleotide intron 7 in *At3g20780* pre-mRNA and that the *hlq* mutation is a null allele. Figure 4B shows the result of an RT–PCR experiment using cDNA templates prepared from wild-type total RNA, *hlq*/+ heterozygote RNA, and *hlq* (–/–) homozygous mutant RNA. This further substantiates the previous results, as an amplicon of ~380bp corresponding to the 300bp predicted intron 7 plus adjacent sequences was seen only in *hlq/+* heterozygote and *hlq* homozygote (–/–) genotypes, and a smaller amplicon of ~ 80bp was seen both in *hlq/+* heterozygotes and control templates plus reverse transcriptase (+RT). Because the size of the wild-type amplicon was larger than the predicted 55bp and only one cDNA clone (AJ297843) has been described (Hartung and Puchta, 2001), it is plausible that an alternative splice donor found 21 nucleotides downstream in intron 7 might be functional, which would add seven amino acid residues to the translated wild-type Top6B.

Despite performing several backcrosses of the original mutant isolate to the parental line to remove extraneous mutations, because of close linkage, 12 other candidate heterozygous SNPs were found in the mapped interval by whole-genome resequencing of *hlq*/+ heterozygotes. This formally raises the possibility that the other mutations might contribute to the pleiotropic *hlq* phenotypes, notwithstanding the fact that none of these SNPs falls within annotated exons or at intron donor–acceptor consensus sites. In order to provide conclusive evidence for a causal link between the *hlq* mutation and the pleiotropic phenotypes, two T-DNA insertion lines were obtained (SALK_140704 and SALK_024455) which disrupt exons 4 and 12 of *At3g20780*, respectively. Segregation of dwarf individuals with pleiotropic defects in root hair abundance and epidermal cell morphology was observed (Supplementary Figs S5, S6 at *JXB* online). Significantly, homozygous mutants of both these lines accumulated anthocyanins in meristems (Supplementary Fig. S5), as seen for *hlq* (Supplementary Fig. S1C). Based on the nomenclature of Schiefelbein and Somerville (1990), *hlq* and the tested T-DNA insertion lines had few normal root hairs of at least 100 μm in length, and sparse spike (non-branched) trichomes (Subramanian *et al.*, 2002) were seen on primary leaves. The initiation of root hairs was normal from the basal end of the trichoblasts (towards the root apex), suggesting that the epidermal cells are not impaired in their ability to determine polarity. This observation is in contrast to the *mid* mutant of the TopoVI regulatory subunit, which has been reported not to express an apical polarity marker ROP2 associated with root hair initiation (Kirik *et al.*, 2007).

**Fig. 5.**
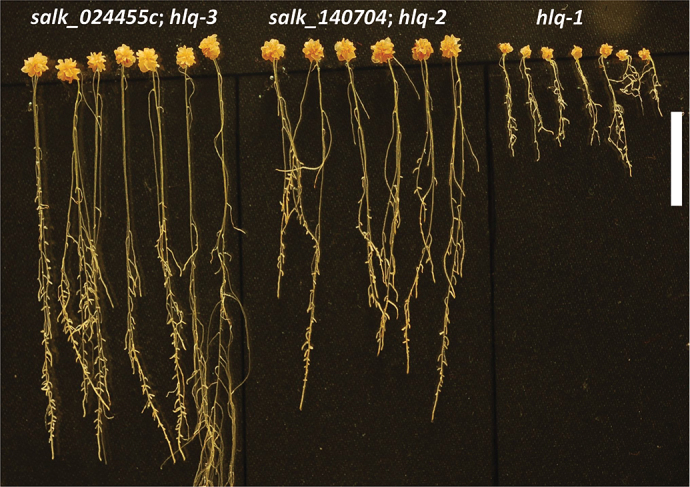
T-DNA knockout lines SALK_140704 and SALK_024455C identify two new hypomorphic alleles of *hlq* based on severity of shoot and root dwarfism and other pleiotropic effects on morphogenesis. Segregating mutants were plated on 0.8% sucrose minimal media and grown for 6 weeks. Bar=1cm.

**Fig. 6.**
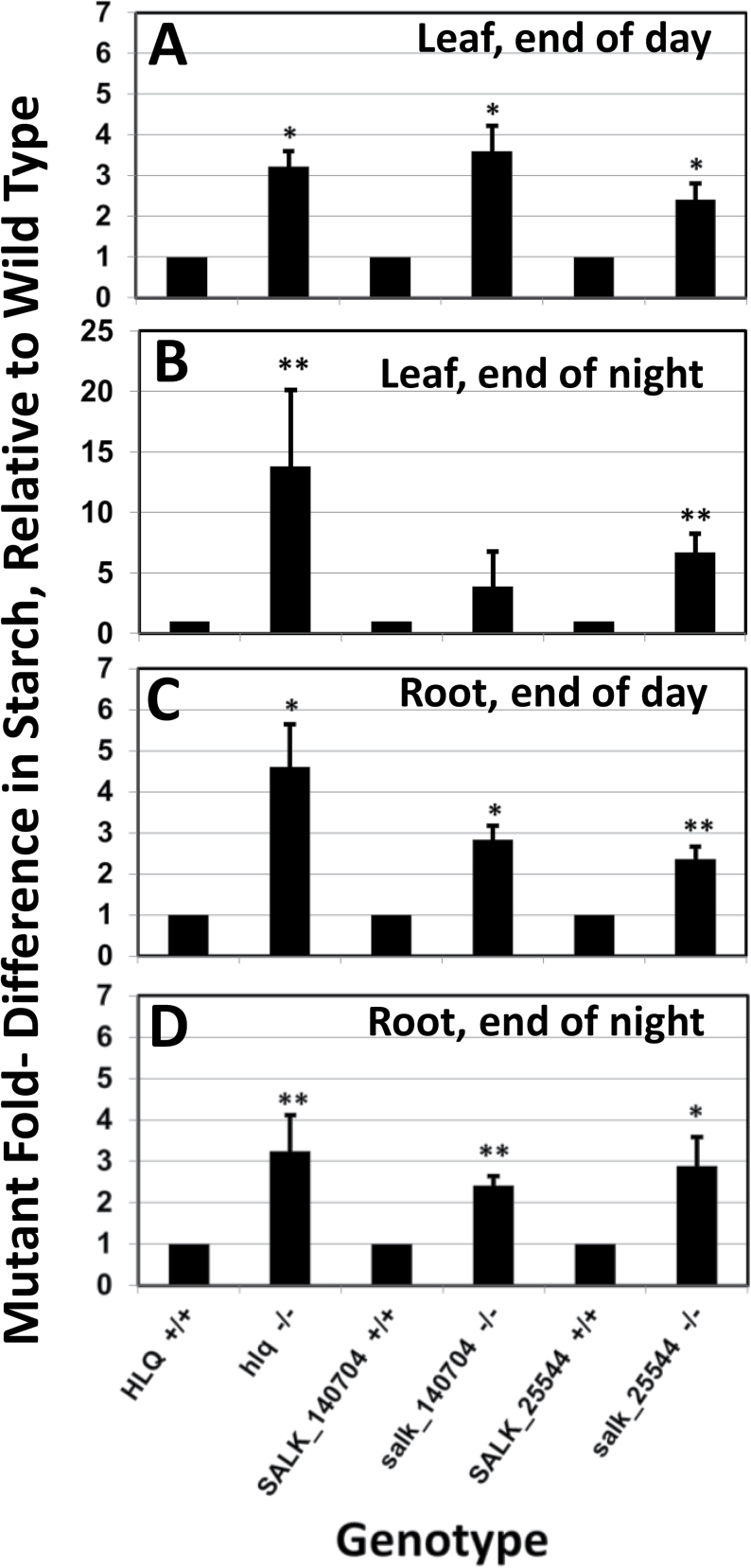
Relative starch accumulation in leaves (A, B) and roots (C, D) of *hlq* mutant alleles compared with isogenic controls at the end of day (A, C) and end of night (B, D) diurnal cycles. Asterisks indicate significantly different from the wild type (*P<0.05; **P<0.01, Student’s paired *t*-test, equal variance assumed). Error bars are the SEM, *n*=4.

Qualitatively the root hairs of *hlq* (not shown) and the T-DNA insertion lines were observed to bulge or branch (Supplementary Fig. S6 at *JXB* online). The roots and shoots of the knockout mutants had a visually rough appearance, and the root differentiation zone and the distal region of the elongation zone were often swollen (Supplementary Figs S5D, S6). Homozygotes of both T-DNA insertion lines had very similar pleiotropic phenotypes to those of *hlq* mutants, but not as severe in terms of dwarfing of roots and shoots (Fig. 5). Interestingly, the presumed homozygous line SALK_024455C was found not to be homozygous, with only a few seeds out of ~60 obtained from the stock centre showing the dwarf phenotype. After verifying heterozygous lines, complementation tests were performed by crossing to *hlq/+* heterozygotes, and non-complementation (predicted 3:1 wild type:mutant segregation) was observed in F_1_ seedlings (Supplementary Fig. S7). PCR genotyping of these individual plants showed that they were heterozygous for the T-DNA insertions, which rules out self-pollination of *hlq*/+ as an alternative interpretation of the results. χ^2^ analysis of the numbers of observed segregating mutants in the F_1_ generation from crosses of heterozygous lines for the T-DNA insertions of SALK_024455C and SALK_140704 with *hlq*/+ provided conclusive evidence that *hlq* is an allele of *BIN3/HYL6/RHL3/TOP6B* (Table 1). Since the T-DNA alleles have not been described previously, they are designated based on decreasing phenotypic dwarf severities (Fig. 5) as *hlq-2/salk_140704* and *hlq-3/salk_024455c*, respectively (Fig. 4C). Analyses of absolute (Table 2) and relative (Fig. 6) starch accumulations in leaves and roots of the *hlq-1*, *hlq-2*, and *hlq-3* mutants compared with isogenic controls validated prior staining results (Fig. 2A, C), and further showed that excess starch accumulation in mutants is not abated during the night, when starch was normally metabolized to give an ~7-fold drop in wild-type controls (Table 2). The strong correlations between fold starch accumulations in mutant leaves (Table 2) or roots (Fig. 6C) taken together with the observed severities of dwarf phenotypes (Fig. 5) supports the interpretation that *hlq-1* is a null allele (Fig. 4A) and that *hlq-2* and *hlq-3* are somewhat leaky or hypomorphic alleles.

**Table 1.** χ^2^ analysis of mutant phenotype segregation (non-complementation) observed in F_1_ progeny of crosses between SALK_24455C/+ and SALK_140704/+ with hlq/+ heterozygous genotypes supports that *HLQ* is an allele of *BIN3/HYP6/RHL3/TOP6B*

Phenotype	Observed SALK_24455^*a*^	Expected^*b*^	Observed SALK_140704^*c*^	Expected^*b*^
Mutant	9	12	47	48.75
Wild type	39	36	148	146.25
Total individuals	48		195	
χ^2^ *P-*value	0.32		0.77	

^*a*^ Six separate crosses.

^*b*^1 mutant:3 wild type segregation null hypothesis for non-complementation, df=1.

^*c*^ Eleven separate crosses.

**Table 2.** Quantitation of starch (mg starch per g FW) in different organs and in response to light in a series of *hlq* mutant alleles

Genotype	Tissue, and time of harvest				Ratio of leaf starch in mutant/wild type (fold effect)	
	Leaf, end of day	Leaf, end of night	Root, end of day	Root, end of night	End of day	End of night
*hlq-1* (–/–) homozygote	15.94	13.56	5.63	4.41	4.1	28.6
HLQ (+/–) heterozygote/WT	3.89	0.47	2.62	1.74		
*salk_140704/hlq-2* (–/–) homozygote	17.18	9.76	2.65	1.72	2.8	12.6
SALK_140704 (+/–) het/WT	6.09	0.78	1.43	1.10		
*salk_25544c/hlq-3* (–/–) homozygote	9.88	9.81	3.25	2.28	1.9	11.9
SALK_25544C (+/–) het/WT	5.14	0.82	1.58	1.06		
Wild type average (±SEM)	5.04±0.64	0.69±0.11	1.88±0.37	1.30±0.22		
Mutant average(±SEM)	14.33±2.25	11.04±1.26	3.84±0.91	2.80±0.82		

### Transcriptome profiling of the *hlq* mutant and meta-analysis of other TopoVI mutant data sets reveals pleiotropy at the levels of chromatin and signalling of light, ROS, hormone, sugar, and calcium affecting carbon metabolism

Since the *HLQ/BIN3/HYL6/RHL3/TOP6B* gene has previously been described as regulating several molecular processes and signalling, it was endeavoured to take a systems approach to better understand the nature of HLQ effects, by analysing deregulated gene expression in *hlq* mutant seedlings using microarrays. The data are provided in Supplementary Datafile 1 at *JXB* online. Of the 25 673 genes on the microarray, ~23% were significantly (*P*<0.05) misregulated in *hlq* compared with control, with 305 and 365 genes being down-regulated and up-regulated, respectively, >2-fold (average *P*<0.002). Using an arbitrary cut-off for fold effects of 1.74 (log_2_=0.8), 514 genes were identified as down-regulated and 559 up-regulated in *hlq* (average *P*<0.003). Supplementary Fig. S8 shows a ‘volcano plot’ for FC of all probes as a function of statistical significance (*P*-values of FC).

The Java desktop application ‘Exploratory Gene Association Networks (EGAN) (Paquette and Tokuyasu, 2010), which provides a contextual graph visualization of transcriptome results, was employed and an intriguing observation was made: excluding 24 cases of gene duplications (where co-regulation is assumed to be due to duplicated regulatory elements), there was a statistically significant chromosomal adjacency for 27 sets/pairs of up-regulated and 24 sets/pairs of down-regulated genes, or ~5% of the 1073 most differentially expressed genes (*P*<10^–21^, χ^2^ test using 1078 ‘control’ genes with average FC symmetrically distributed about zero; Supplementary Table S2 at *JXB* online). This number of gene pairs was more than three times the number of cases that would be predicted based on observed discordant pairs (14 pairs of adjacent differentially expressed genes that had opposite signs for FC, which fit well to a binomial model of chance occurrence). This observation suggested that the TopoVI complex containing HLQ/TOP6B acts in part by chromatin remodelling of proximal clusters of two and three genes, as has been observed for the differentiation of haematopoietic stem cells to erythroid and neutrophil cell types (Kosak *et al.*, 2007). Consistent with this interpretation is that 56% of those immediately adjacent co-regulated genes are encoded on opposite strands, ruling out co-transcriptional control *per se* (e.g. by read-through of RNA polymerase II). The average distance between the distal ends of the co-regulated genes was 8 kbp (Supplementary Table S2). Previous studies showed that the TopoIIα-like RHL1 and BIN4/MID proteins bind each other, TOP6A (but not TOP6B), and DNA (Breuer *et al.*, 2007; Sugimoto-Shirasu *et al.*, 2005). It is of note that Simkova *et al.* (2012) showed by chromatin immunoprecipitation that *Arabidopsis* TopoVI subunits RHL1 and TOP6A directly bind to the promoter/transcription initiation sites of a pair of adjacent genes encoding unknown domains, *At1g24145* and *At1g24147*, claiming that the TopoVI complex functions directly in initiation and elongation of transcription for different classes of genes subject to regulation by singlet and peroxide pathways of ROS.

Given the recent claim that TOP6A/CAA39/AtSPO11-3/RHL2/BIN5 is an integrator of singlet oxygen and H_2_O_2_ stress response pathways and therefore regulates expression of several thousand genes in *Arabidopsis* (Gadjev *et al.*, 2006), it was first sought to compare those reported ^1^O_2_- and H_2_O_2_-responsive genes subject to regulation by TOP6A/CAA39 (Simkova *et al.*, 2012) with first-generation transcriptome microarray studies on *bin5/top6a* and *bin3/top6b* (Yin *et al.*, 2002) as a framework for interpretation of the transcriptome results with *hlq/top6b*. Of the 255 genes claimed to be down-regulated in the *caa39/top6a* mutant, 13 genes (or only 5%) were strictly concordant with the 314 genes claimed to be down-regulated in *bin5/top6a* (Supplementary Table S3 at *JXB* online) (but with the caveats that *bin5* results were from a smaller number of genes interrogated by the Affymetrix 8.3K GeneChip^®^, and *caa39* is claimed to be a weak allele). This observation raised questions about interpretations of specificity drawn by previous microarray studies of TopoVI mutants.

Transfer from dark to light of 5-day-old *top6a/caa39* mutants in the *fluorescent in blue light* (*flu*) mutant background triggers photo-oxidative stress, resulting in misexpression of 1093 genes (Simkova *et al.*, 2012), similar to the number of significantly misregulated genes that were observed in *hlq*. This situation permits a reasonable analysis for agreement across experiments. Supplementary Table S3 at *JXB* online provides a comparison of genes displaying altered transcript levels for *top6a/caa39* (Simkova *et al.*, 2012) with the present results for *hlq/top6b*. For the 51 genes (cluster 1) reported to be up-regulated in *top6a/caa39* mutants unrelated to singlet oxygen signalling (and enriched for genes related to DNA repair), 13 were significantly up-regulated in *hlq* and one was significantly down-regulated; this is a very good agreement in terms of qualitative effect, but represents only about a quarter of the results that would be expected, assuming reproducibility.

For the 113 genes claimed to be induced by ^1^O_2_ in a TopoVI-dependent manner (cluster 4), 22 genes were observed to be significantly up-regulated and 12 genes significantly down-regulated, for an overall concordance of 30% differential expression, but not strictly (the null hypothesis of reproducibility was down-regulation in *hlq*, but more genes were up-regulated). For the cluster of 55 genes hyperactivated by ^1^O_2_ in *top6a/caa39* mutants (cluster 5, indicated as TopoVI-repressed), 12 up-regulated and two down-regulated genes were observed in *hlq*, a similar concordance (25%) with the TopoVI-specific effects described above. Similarly for cluster 2, in which were indicated 25 down-regulated genes in *top6a/caa39* not subject to singlet oxygen regulation, four down-regulated and two up-regulated genes were observed. This generally fits the null hypothesis, but with a low concordance (24%) as observed for the singlet oxygen-specific genes claimed to interact with *top6a/caa39*. For the 199 genes (clusters 6 and 8) indicated as singlet oxygen inducible and up-regulated in *top6a/caa39* (i.e. indicated to be TopoVI repressible), good concordance was observed with only five down-regulated genes and 90 up-regulated genes (48% of the total).

Turning attention to further data sets, an analysis of ~5500 genes in which 316 genes were reported as down-regulated in both *bin3/top6b* and *bin5/top6a* mutants (Yin *et al.*, 2002) was examined. Supplementary Table S3 at *JXB* online provides the *hlq*/WT log_2_FC results for genes identified in that list. Good concordance was observed, with 137 genes being significantly misregulated in the *hlq/top6b* mutant (43%), and very good reproducibility, with only 6% (20) of these genes being up-regulated and 117 genes down-regulated in *hlq*, as hypothesized. Taken together, this meta-analysis supports the hypothesis that TopoVI mutants reproducibly alter expression of specific gene sets that can provide clues to the underlying cross-talk in gene networks controlling growth and development.

Finally, those genes (clusters 3 and 7) reported as down- and up-regulated, respectively, by singlet oxygen produced after a shift to high light conditions in the *flu* background, but which were claimed not to interact with the *top6a/caa39* genotype (Simkova *et al.*, 2012), were compared. In this case, the null hypothesis in the meta-analysis is non-concordance (low reproducibility) for the *hlq/top6b*-specific effects. Of the 203 genes reported as down-regulated by a shift to high light, 39 were significantly up-regulated in *hlq*, whereas 24 genes were significantly down-regulated (total concordance 31%), similar to or higher than the overall percentage seen for the observed concordance for TopoVI-specific effects (clusters 1, 2, 4, 5, 6, and 8).

Similarly, for cluster 7 (365 genes up-regulated by light shift but independent of *top6a/caa39*), 132 significantly up-regulated and 24 down-regulated genes were observed in *hlq/top6b*, a very good agreement of 43% overall and strict reproducibility in terms of expected up-regulation. Taken together, these results showing high *hlq/top6b* concordance, vis-à-vis claimed *top6a/caa39* non-specific effects, suggest that the TopoVI modes of action include pathways other than the oxidative stress response. Despite the relatively good agreement for differentially expressed genes observed between the present results and two independent experiments that reported similar numbers (several hundred) of TopoVI-dependent down-regulated genes, meta-analysis across all three microarray experiments found only seven consistently misregulated genes, and strict concordance was marginal (Table 3). This finding reinforces the notion that the pleiotropic nature of TopoVI mutants defies easy descriptions of function, which raises questions of whether TopoVI is indeed a key integrator of the principal pathways of brassinosteroids (Yin *et al.*, 2002), cell cycle control (Hartung *et al.*, 2002; Sugimoto-Shirasu *et al.*, 2002), or singlet oxygen (Simkova *et al.*, 2012) signalling as previously claimed. Since *hlq* came out of a screen for misregulated ABA-inducible gene expression (Subramanian *et al.*, 2002), TopoVI activity probably targets many different signalling pathways and processes, either directly or indirectly.

**Table 3.** Genes significantly misregulated in three independent microarray experiments^*a*^ in TopoVI mutants

AGI	Annotation, gene ontology process/function	*top6a/caa39* ^*b*^	*hlq/top6b* log_2_FC (this work)	Concordance?
At5g42650	CYP74A/DELAYED DEHISCENCE2; allene oxide synthase	Singlet induction TOP6A dependent	–0.67	Yes
At5g47370	HAT2, homeodomain transcription factor induced by auxin; root morphogenesis related	Singlet repressed, TOP6A independent	–0.29	Yes
At5g57560^*c*^	TOUCH4/cell wall modifying; rapidly induced by environmental stimuli	TOP6A repressed, independent of singlet induction	1.29	Not for *bin3*
At1g01120	KCS1, 3-ketoacyl-CoA synthase, critical for fatty acid elongation in wax biosynthesis	Singlet induced, TOP6A independent	–0.49	Not for singlet
At2g44940	DREB subfamily A-4 of Ethylene Response Factor/APETELA2 domain transcription factor	Singlet induced, TOP6A independent	–0.49	Not for singlet
At4g20780	CALMODULIN LIKE 42, EF hand domain calcium and protein binding; involved in trichome branching, abiotic stress responses	Singlet induced, TOP6A independent	–0.27	Not for singlet
At2g17880	Chaperone DnaJ heat shock protein, response to sugar	Singlet repressed, TOP6A independent	0.25	No

^*a*^ Originally reported (Yin *et al.*, 2002) as down-regulated >2-fold in both *bin3/top6b* and *bin5/top6a* mutants.

^*b*^ Genes classified (Simkova *et al.*, 2012) as ‘*topo6a/caa39* induced’ (cluster 1); ‘*topo6a* repressed’ (cluster 2); ‘singlet repressed, TOP6A independent’ (cluster 3); ‘singlet induced TOP6A dependent’ (cluster 4); ‘singlet induced TOP6A repressed’ (cluster 5); ‘TOP6A repressed, independent of singlet induction’ (clusters 6, 8); ‘singlet induced, TOP6A independent’ (cluster 7).

^*c*^ Reported (Yin *et al.*, 2002) as down-regulated; brassinosteroid induction dependent on BIN3/TOP6B and BIN5/TOP6A.

Given the pleiotropic nature of *hlq*, MAPMAN software (Usadel *et al.*, 2005) (http://mapman.gabipd.org) was used to analyse empirically the transcriptome data set in terms of metabolic and regulatory pathways affected in *hlq* mutants. Supplementary Fig. S9 and Supplementary Datafile 1 at *JXB* online provide, in the form of a relative heat map and lists, respectively, the number and degree of 2575 metabolism-related genes that are significantly misregulated in *hlq* (average *P*<0.005 for visibly coloured genes in Supplementary Fig. S9). Genes for photosynthesis, photorespiration, terpenoid biosynthesis, nitrogen assimilation, tetrapyrrole/chlorophyll/haem/phytochrome chromophore biosynthesis, the reductive pentose phosphate Calvin/Benson/Bassham cycle, and starch biosynthesis were uniformly down-regulated in *hlq* seedlings. This generally correlated with observed chlorosis in the mutant (Fig. 5), but was inversely correlated with starch accumulation (Figs 2, 6), an unexpected finding that suggests feedback regulation of carbon metabolism. Supplementary Datafile 2 documents the findings of specific genes, pathways, and processes significantly altered at the transcriptome level in *hlq*.

It was of particular interest that one of the down- regulated (2.38 FC, *P*<0.00001) tetrapyrrole genes is *At5g13630/GENOMES UNCOUPLED5*/*Mg-CHELATASE-H*, encoding an enzyme of chlorophyll biosynthesis variously described as involved in plastid to nucleus retrograde signalling (Mochizuki *et al.*, 2001; Susek *et al.*, 1993) and a mediator of ABA signal transduction, possibly as an ABA receptor modulating expression of WRKY-domain transcriptional repressors, ROS homeostasis, and lipid β-oxidation (Shen *et al.*, 2006; Wu *et al.*, 2009; Shang *et al.*, 2010; Jiang *et al.*, 2011; Tsuzuki *et al.*, 2011; Xu *et al.*, 2012). Remarkably, the downstream targets of GUN5, *At2g33150/3-ketoacyl-CoA thiolase/KAT2* and *At1g80840/WRKY40*, are implicated in ABA signalling (Shang *et al.*, 2010; Jiang *et al.*, 2011; Liu *et al.*, 2012), and both are significantly up-regulated in *hlq* mutants (2.96 FC, *P*<0.0001; and 1.96 FC, *P*<0.002, respectively (Table 4). *KAT2* is categorized in Supplementary Fig. S9 at *JXB* online as involved in branched-chain amino acid catabolism, which was over-represented with significantly down-regulated biosynthetic genes and up-regulated catabolic genes (*P*=0.003: Supplementary Datafile 1). Interestingly, several of the *WRKY* genes up-regulated in *hlq* (Supplementary Table S4) are also expressed specifically in trichomes (*WRKY8*, *-15*, *-18*, and *-33*) (Jakoby *et al.*, 2008) and involved in hormone signalling cross-talk and oxidative stress response (*WRKY25*) (Hu and Ma, 2006). Similar to mutants that do not produce mature trichomes (Marks *et al.*, 2009), many abiotic stress effectors were misregulated in *hlq* (Supplementary Datafile 1), including genes for ABA biosynthesis and metabolism, members of the *PYRABACTIN-RESISTANT1/REGULATORY COMPONENTS OF ABA RECEPTOR11/RCAR11* class of ABA receptors, and genes with functional evidence as effectors of ABA signalling and biosynthesis (Table 4).

**Table 4.** ABA biosynthesis and signalling genes with significantly altered expression in *hlq* mutant seedlings

AGI	Gene name	Annotation	*hlq*/WT log_2_FC	*P*-value
At1g15520	*ABCG40*	ABC transporter; ABA importer	1.91	0.0001
At2g47130	*SDR3*	Short-chain dehydrogenase/reductase ABA2-like	1.73	0.0001
At2g33150	*KAT2/PED1*	Peroxisomal 3-ketoacyl-CoA thiolase, fatty acid β-oxidation; positive ABA effector	1.56	0.0001
At1g80840	*WRKY40*	Pathogen-induced transcription factor; ABA repressor	0.97	0.001
At5g67300	*MYB44*	R2R3 MYB transcription factor; ABA sensitivity	0.85	0.01
At1g56070	*LOS1*	Low response to Osmotic Stress1; translation elongation factor 2-like; cold stress response	0.52	0.001
At5g58670	*PLC1*	Phospholipase C1; ABA, drought, salt, cold response	0.45	0.05
At4g26080	*ABI1*	ABA insensitive1; protein phosphatase 2C	0.35	0.008
At3g57530	*CPK32*	Calcium-dependent protein kinase32; phosphorylates ABA RESPONSE FACTOR4	0.31	0.01
At1g33560	*ADR1*	ACTIVATED DISEASE RESISTANCE1; NBS-LRR; interacts with ABI1 in drought response	0.31	0.03
At1g69260	*AFP1*	ABI5-Binding Protein; domain unknown function	0.28	0.03
At3g24650	*ABI3*	ABA insensitive3; B3 domain transcription factor	0.26	0.02
At2g40220	*ABI4*	ABA insensitive4; AP2 domain transcription factor	0.23	0.03
At4g01026	*PYL7*	Pyrabactin-Like7; ABA receptor	0.22	0.04
At5g45870	*PYL12*	Pyrabactin-Like12; ABA receptor	0.22	0.05
At3g25010	*RLP41*	Receptor Like Protein41; ABA hypersensitive to chlorosis	–0.21	0.04
At2g32860	*LOS15*	AtBGL2; Beta glucosidase33; ABA-GE hydrolase	–0.24	0.02
At1g80080	*TMM/RPL17*	Too Many Mouths; stomatal development; ABA insensitive to chlorosis	–0.26	0.02
At1g35515	*HOS10/MYB8*	High response to Osmotic Stress10; ABA hypersensitive	–0.33	0.02
At5g45340	*CYP707A3*	ABA 8’-hydroxylase; phaseic acid synthesis	–0.46	0.03
At1g73000	*PYL3*	Pyrabactin-Like3; ABA receptor	–0.46	0.02
At4g17870	*PYR1*	Pyrabactin Resistent1; ABA receptor	–0.58	0.01
At5g53160	*PYL8*	Pyrabactin-Like8; ABA receptor	–0.63	0.0008
At3g63520	*NCED1*	Nine-*cis*-epoxycarotenoid dioxygenase	–0.75	0.0005
At5g67030	*ABA1*	Zeaxanthin epoxidase; mutant ABA deficient	–0.84	0.002
At4g15560	*CLA1*	1-Deoxyxylulose 5-phosphate synthase; chloroplastos alterados1; ABA deficient	–0.87	0.0002
At1g52400	*AtBG1*	Beta glucosidase18; ABA-GE hydrolase	–1.01	0.0002
At2g41070	*DPBF4/EEL*	Dc3-Pro Binding Fctr4; bZIP; Enhanced Em Level	–1.24	0.0002
At1g64670	*CED1*	9-CIS EPOXYCAROTENOID DIOXYGENASE DEFECTIVE; BODYGUARD1; epidermal wax biosynthesis; alpha-beta hydrolase	–1.60	0.0008

Supporting the observed phenotypes of lignin and anthocyanin accumulation in *hlq* (Fig. 3A, B; Supplementary Figs S1C, S5D at *JXB* online), many genes involved in aromatic amino acid and phenylpropanoid/lignin biosynthesis were up-regulated in *hlq*, whereas flavonoid biosynthetic genes were both up- and down-regulated (Supplementary Fig. S9; Supplementary Datafile 1), some of which are mono-oxygenases that have haem cofactors (see above). The strong up-regulation of *NITRILASE2*, which functions in an alternative pathway of wound- and pathogen-induced auxin (IAA) biosynthesis from indole-3-acetonitrile (Huh *et al.*, 2012) and degradation of tryptophan-derived glucosinolates, indicates that auxin or tryptophan amino acid homeostasis may be affected in *hlq*, or that the *hlq* defect in trichome development may be responsible for the observed (Supplementary Fig. S9) misregulation of glucosinolate biosynthetic genes (Jakoby *et al.*, 2008). Since trichome-specific expression of the cell cycle checkpoint CYCLINB1;2 results in rescue of the *top6a/rhl2* and *mid* mutant trichome defects but not of their dwarf phenotypes (Kirik *et al.*, 2007), it is likely that the misregulation of glucosinolate pathways in *hlq* mutants is a direct effect of TopoVI on carbon partitioning processes.

With regards to the pleiotropic cell wall phenotypes of *hlq*, several genes encoding proteins (EXPANSINS, ARABINOGALACTAN PROTEINS) that modify or contribute to wall structure and/or metabolism were up-regulated (Table 5), providing correlative evidence for the functions of those genes in the cell wall phenotypes of *hlq*, including the increased abundance of pectins (Fig. 3E) and radial swelling of epidermal cells (Fig. 2C), which was phenocopied by the *N*-glycosylation inhibitor tunicamycin (Supplementary Fig. S3A, B at *JXB* online). Arabinogalactan proteins are involved in root and stem expansion (Willats and Knox, 1996; Ding and Zhu, 1997; Park *et al.*, 2003) and broadly implicated in plant development including programmed cell death (Gao and Showalter, 1999) similar to the observed *hlq* phenotype, and possibly in ABA-inducible gene expression (Desikan *et al.*, 1999).

**Table 5.** List of cell wall genes most up- and down-regulated in *hlq* seedlings by transcriptome profiling

AGI	Annotation	*hlq/*WT log_2_FC	*P*-value
Up-regulated wall biosynthesis			
At2g18660	EXPANSIN-LIKE B3	3.32	<0.0002
At3g45970	EXPANSIN-LIKE A2	1.29	<0.0007
At2g22470	ARABINOGALACTAN PROTEIN 2	2.21	<0.0002
At1g35230	ARABINOGALACTAN PROTEIN 5	1.14	<0.0007
At4g09030	ARABINOGALACTAN PROTEIN 10	1.07	<0.0003
At5g64310	ARABINOGALACTAN PROTEIN 1	1.00	0.002
At2g45220	PECTINESTERASE	2.73	<0.0009
At3g48580	ENDO-XYLOGUCAN TRANSFERASE	1.20	<0.0003
At5g57560	XYLOGUCAN TRANSFERASE/TOUCH4	1.29	<0.0002
At4g23990	CELLULOSE SYNTHASE/CSLG3	0.84	<0.0006
At3g02230	UDP-l-Ara MUTASE/RGP1^*a*^	0.94	<0.0006
At4g25810	XYLOGUCAN ENDO-TRANSGLYCOSYLASE6/XTR6	1.06	<0.0004
At3g07160	GSL10; GLUCAN SYNTHASE-LIKE10, β-1,3 CALLOSE	0.35	<0.006
Up-regulated wall catabolism			
At3g57510	POLYGALACTURONASE/ADPG1	1.42	<0.02
At4g13210	PECTATE LYASE	1.06	<0.0003
At4g30270	MANNAN-XYLOSE HYDROLASE/MERISTEM5B	1.07	<0.0003
At5g20950	β-1,4-GLUCANASE	0.90	<0.0008
Down-regulated wall metabolism			
At2g06850	ENDO-XYLOGUCAN TRANSFERASE/EXGT-A1	–1.43	<0.0006
At4g37800	ENDO-XYLOGUCAN TRANSFERASE	–1.20	<0.0004
At5g39310	EXPANSIN 24	–0.92	<0.002
At2g37640	EXPANSIN A3	–0.73	<0.002
At4g28250	EXPANSIN B3	–0.72	<0.003

^*a*^ Annotated in Rautengarten *et al.* (2011). RGP1, REVERSIBLY GLYCOSYLATED POLYPEPTIDE1.

### Characterization of *hlq* whole cell wall composition by infrared spectroscopy and quantitation of glycosyl composition

**Fig. 7.**
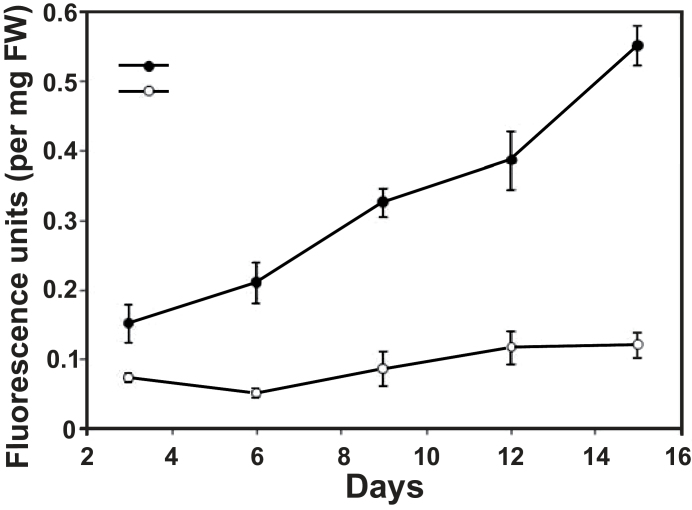
Callose content of wild-type versus *hlq* during growth of seedlings. Mutants of *hlq* had five times more callose than the wild type after 15 d of growth. Error bars represent ±SEM of three replicates. The results presented are for the *abi2-1/hlq* double mutant; however, the phenotypes described were independently verified on Ler/*hlq* single mutants.

The metabolism of callose (β-1,3-glucan) within plant cell walls is essential to many developmental, physiological, and stress-related processes (Chen and Kim, 2009). Production of callose is induced by mechanical stress and wounding, and under conditions of cellulose deficiency (e.g. in cell wall mutants). Some dwarf mutants ectopically deposit callose (Lukowitz *et al.*, 2001), pectin (His *et al.*, 2001), and the phenolic polymers suberin (Cheng *et al.*, 2000) and lignin (Burk *et al.*, 2001), thereby compromising the stability and flexibility of the cell wall. Treatment of wild-type plants with tunicamycin resulted in the appearance of patches of callose accumulation within the root elongation zone (Supplementary Fig. S3D at *JXB* online), similar to the phenotype of *hlq* mutants (Subramanian *et al.*, 2002). A comparison of callose content in *hlq* and the wild type during growth on sucrose-supplemented agar media indicated that the *hlq* mutants showed a steady increase in callose accumulation over 15 d, with mutants accumulating up to five times the callose content of wild-type plants (Fig. 7).

Yariv reagent binds arabinogalactan proteins and triggers accumulation of callose by induction of the callose synthase gene *At3g07160/GLUCAN-SYNTHASE-LIKE10* and the transcription factor gene *WRKY40* (Guan and Nothnagel, 2004), which were both significantly up-regulated in *hlq* (Tables 4, 5). Similarly, the transcription factor gene *MYB44* was up-regulated in *hlq* (Table 4) and has been shown to regulate callose production in *Arabidopsis* (Lü *et al.*, 2011). Since the present results suggested that *hlq* mutants have a generalized perturbation in cell wall composition, *hlq* mutant cell walls were characterized by spectroscopic and biochemical methods.

**Fig. 8.**
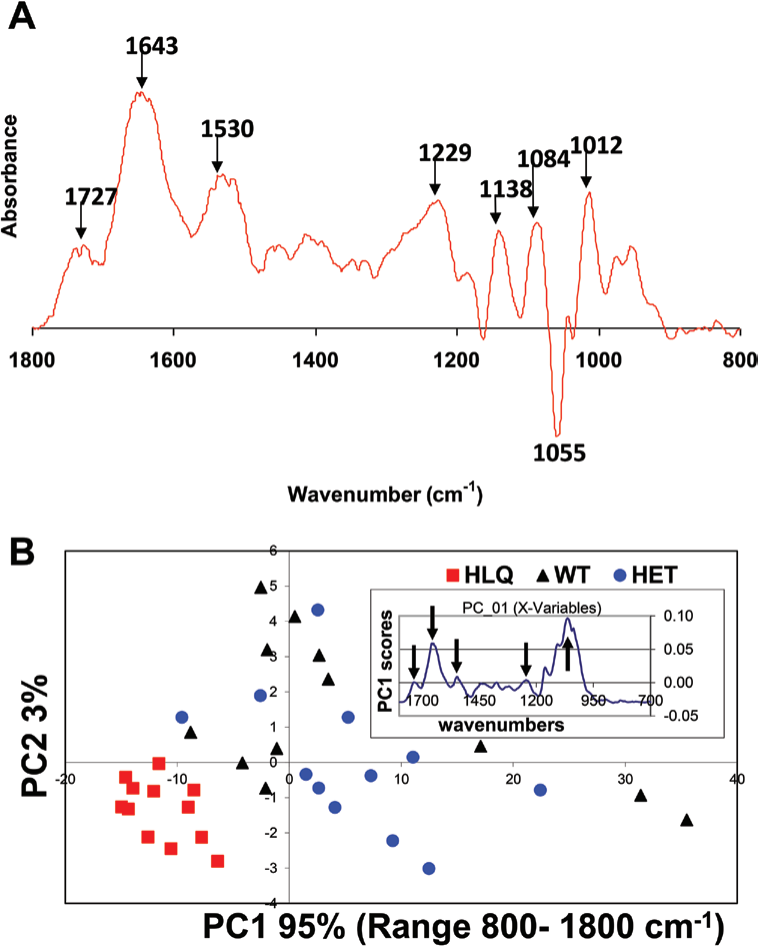
Fourier transform infrared (FTIR) microspectroscopy analysis of dehydrated cell wall extracts of *hlq* and the wild type. (A) Digital subtraction frequency–structure correlation chart. Difference (Δ) spectrum of *hlq* minus wild-type FTIR spectra. The peak at wavenumber 1727cm^–1^ corresponds to the C=O stretch of esterified pectins; peaks at 1643cm^–1^ (C=O stretch), 1530cm^–1^ (N-H amide), and 1229cm^–1^ (C-N stretch) correspond to secondary amide bonds attributed to cell wall peptides, or lignin for 1643cm^–1^ (McCann *et al.*, 1992); peaks at 1138cm^–1^ and 1084cm^–1^ are probably due to the C-O-C glycosidic bonds of various polysaccharides which differ between *hlq* and the wild type; the negative peak at 1055cm^–1^ is the ring C-O of cellulose, and 1010cm^–1^ is probably the ring C-C. (B) Principal component analysis of FTIR spectra separates the *hlq* and controls (WT and heterozygote samples) into two non-overlapping clusters which account for 98% of the variation. Inset: plot of the PC1 loadings versus wavenumber, showing that wavenumbers 1735, 1657, 1546, and 1244cm^–1^ (esterified pectins, lignin/peptides) are dominants in addition to cellulose at 1063cm^–1^ (arrows).

Along with soluble expansins and structural proteins including hydroxyproline-rich extensins and arabinogalactan proteins, four primary and secondary polymer classes make up the plant cell wall: cellulose, hemicellulose, pectin, and lignin. FTIR microspectroscopy, which measures the energy of asymmetric molecular bond vibrations, is a rapid, non-invasive method for detecting a range of polysaccharides affecting cell wall architecture *in muro*, based on their functional groups, including, but not limited to, carboxylic esters, phenolic esters, protein amides, and carboxylic acids. A digital subtraction plot of the *hlq* FTIR spectrum minus that of the wild-type spectrum from extracted cell walls is provided in Fig. 8A. The frequency–structure peak at wavenumber 1727cm^–1^ corresponds to the C=O stretch of esterified pectins, consistent with the qualitative result of pectin staining showing elevated pectins in *hlq* leaves (Fig. 3E) (Alonso-Simon *et al.*, 2011). Secondary amide bonds attributed to cell wall peptides (McCann *et al.*, 1992) were observed at 1643cm^–1^ corresponding to the C=O stretch, at 1530cm^–1^ for the N-H amide, and at 1229cm^–1^ for the C-N stretch. The peaks observed at 1138cm^–1^ and 1084cm^–1^ are probably due to the C-O-C glycosidic bonds of various polysaccharides that differ between *hlq* and the wild type. Importantly, the negative peak at 1055cm^–1^ probably corresponds to the ring C-O of cellulose (Alonso-Simon *et al.*, 2011) and homogalacturonan, whereas the positive peak at 1010cm^–1^ probably represents the ring C-C stretch of various polysaccharides (pectins). These results suggest that there is a deficiency of cellulose or hemicelluloses and an increased abundance of pectins in the *hlq* mutant as compared with the wild type.

The results of PCA discriminatory analysis on the *hlq* and wild-type wall spectra are shown in Fig. 8B. Most of the variation (98%) in FTIR spectral differences between cell wall extracts of *hlq*, *hlq*/+ heterozygotes, and wild-type walls could be attributed to two PCs, with replicate *hlq* homozygous samples clustering separately in the lower left quadrant from *hlq/+* heterozygotes, and wild-type samples clustering broadly together in the other three quadrants (Fig. 8B). Plotting the PC that accounted for 95% of the variation as a function of wavenumber (Fig. 8B, inset arrows) showed peaks at 1735cm^–1^ (esterified pectins), 1657cm^–1^ (lignin), 1546cm^–1^ (secondary peptide amide), 1244cm^–1^ (C-N peptide amide), and 1063cm^–1^ (cellulose or homogalacturonan), corresponding well to previous reports (McCann *et al.*, 1992). It is concluded from this analysis that the *hlq* mutant is deficient in cellulose or hemicelluloses, or both, and has elevated levels of pectins, lignins, and cell wall proteins. This conclusion was further supported by the microarray results for cell wall structural proteins EXPANSIN-LIKE and arabinogalactan proteins (Table 5).

**Fig. 9.**
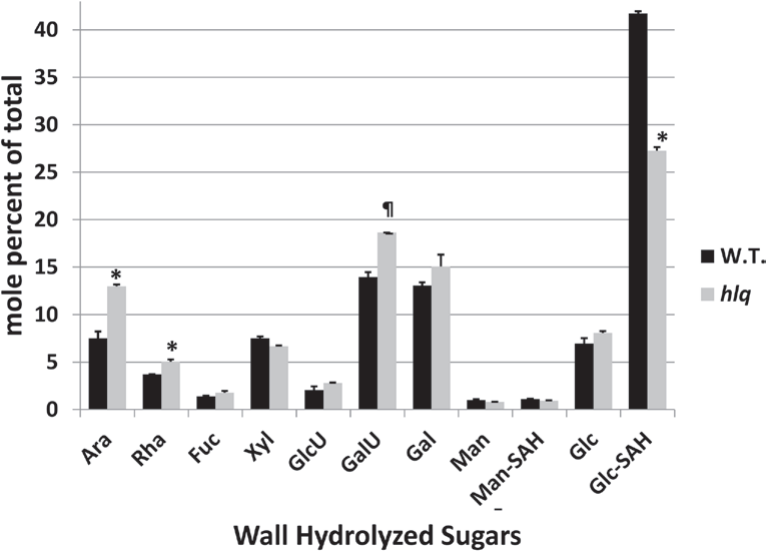
Glycosyl composition (mol%) of the whole cell wall fraction from wild type (WT) and *hlq* mutant *Arabidopsis* seedlings, without and with prior H_2_SO_4_ swelling and hydrolysis (SAH) to differentiate cellulose and tightly bound mannosyl polymers from extractable (e.g. hemicellulose) polysaccharides. Cell walls of *hlq* had significantly decreased cellulose and compensatory increases in arabinosyl, rhamnosyl, and galactosyl residues typical of pectic arabinogalactans. Sugar residues were measured as TMS methylglycosides by GC-FID after methanolysis of whole walls (before and after SAH). Glc-SAH and Man-SAH represent the additional residues detected due to SAH. Error bars indicate ±SEM (*n*=2). Asterisks (*) indicate significantly different from both wild-type and *hlq/+* heterozygote samples, *P*<0.02 (Student’s two-sided *t*-test, equal variance assumed). ¶ indicates significantly different from wild-type samples, *P*<0.02.

In order to differentiate between the effects of *hlq* on cellulose and hemicellulose levels, the glycosyl composition of whole cell wall extracts subjected to methanolysis and trimethylsilylation combined with GC with or without prior swelling and hydrolysis of cellulose (SAH) using concentrated sulphuric acid was analysed. On a mole percentage basis of constituent sugar residues, the whole cell wall fraction of *hlq* seedlings contained significantly more abundant components of pectic arabinogalactans than the wild type, in particular arabinose (5.5 mol% increase), rhamnose (1.3 mol% increase), and galacturonic acid (4.6 mol% increase), whereas *hlq* mutant walls contained significantly less cellulosic glucose (–14.5 mol% decrease) than those of the wild type (Fig. 9). This supports the finding that *hlq* accumulates more pectin in leaves than the wild type (Fig. 3E) and has a wall architecture deficient in cellulose (Fig. 8), and the conclusion that cellulose deficiency and/or pectin overabundance resulted in severely compromised epidermal cell wall structural integrity (Fig. 2C; Supplementary Figs S1H, S6 at *JXB* online).

## Discussion

Although previous claims (Hartung *et al.*, 2002; Sugimoto-Shirasu *et al.*, 2002; Schrader *et al.*, 2013) on the role of TopoVI, found only in plants and archaebacteria, in the decatenation of endoreplicated chromosomes during cell growth are not under debate, based on the results obtained on *hlq* mutants, a more complex role for TopoVI in transducing at the level of chromatin environmental and internal signals controlling growth and development is proposed. Others have also recognized this possibility (Sugimoto-Shirasu *et al.*, 2002; Yin *et al.*, 2002; Breuer *et al.*, 2007; Kirik *et al.*, 2007). The pleiotropic phenotypes of *hlq* were previously characterized, in particular that mutants are skotomorphogenic, with deformed epidermal cells which stain for callose, abnormal and reduced root hairs and leaf trichomes, and stochastic ectopic expression of an ABA- and auxin-inducible transgene. Here *hlq* mutants were further characterized at the physiological and transcriptome levels as accumulating starch, callose, and anthocyanins, with cell walls abundant in pectins, lignin, and cell wall proteins. Moreover, despite the absence of endoreplication in *Arabidopsis* inflorescences (Galbraith *et al.*, 1991), it was shown here (Supplementary Fig. S1B, F at *JXB* online), as has been shown by others (Sugimoto-Shirasu *et al.*, 2002), that *hlq/top6b* mutants can produce mostly normal flowers but neither homozygous male nor female *hlq* sporophyte organs functioned properly in reproduction, notwithstanding artificial nutrient supplementation or the previous claims that the *hyp6/top6b*, *rhl2/top6a/atspo11-3,* and *bin3-1* alleles are null yet produce viable seeds (Sugimoto-Shirasu *et al.*, 2002; Yin *et al.*, 2002). Consistent with the notion that TopoVI is important for reproduction is the observation that in rice the TopoVI subunits are expressed maximally in pre-pollinated flowers (Jain *et al.*, 2006). Here *hlq* and two additional alleles that vary in their phenotypic severity were isolated and it was shown that they are alleles of *bin3/hyp6/rhl3/*Topoisomerase 6B.

The *rhl3* allele of *TOP6B* is documented in TAIR (http://www.arabidopsis.org) as a mutation of the splice acceptor in intron 17, which is stated to result in the transcription of an mRNA with at least three altered sizes that create a premature stop codon in the last exon. Hartung *et al.* (2002) described an unnamed T-DNA mutant allele that disrupted exon 12 and deleted 268bp of the *TOP6B* gene including exon13. On the basis of root calli death 3 weeks after growth induction, they argued that deficiency of AtTOP6B results in a general growth defect. Because of the absence, in the present case, of a correctly spliced intron 7 mRNA (Fig. 4) and based on the prediction that the *hlq* mutation sits adjacent to a highly conserved aliphatic residue two residues distal to the GXG motif of the ATP binding site in the ATPase-like domain (Corbett and Berger, 2006), it is argued here the *hlq-1* mutation is a *bona fide* null allele. Phenotypic analysis of two new hypomorophic *hlq* alleles (Fig. 5) supports this contention. The various descriptions by others of presumed null alleles demonstrate that, due to the pleiotropic nature of *top6B* mutants, limited phenotypic characterizations can give misleading interpretations of gene function. This notion is supported by the contradictory reports of *bin3/hyp6/top6b* mutant effects on chromosome ploidy levels for several cell types (Hartung *et al.*, 2002; Sugimoto-Shirasu *et al.*, 2002; Yin *et al.*, 2002) and the broad lack of concordance from meta-analysis of the *hlq* transcriptome with other previously published TopoVI mutant transcriptomes (Table 3; Supplementary Table S3 at *JXB* online), including other claimed null alleles.

The *Arabidopsis* genome encodes a second type II topoisomerase (*At3g23890*) that is differentially regulated from TopoVI (Breuer *et al.*, 2007) and probably functions in mitosis, T-DNA integration, and cell cycle regulation (Xie and Lam, 1994; Makarevitch and Somers, 2006), and may have some overlapping functions with TopoVI (Sugimoto-Shirasu *et al.*, 2005). The claims that BIN4/MID, a DNA-binding component of TopoVI, functions primarily as a DNA damage response effector during post-mitotic endocycles (Breuer *et al.*, 2007) and transcriptional silencing (Kirik *et al.*, 2007) mediated through the cell cycle checkpoint effector Ataxia Telangiectasia-mutated and Rad3-related (*At5g40820/ATR*) and ectopically expressed markers *At2g31320/poly* (*ADP-ribose*) *polymerase1/PARP1*, *At4g02390/PARP2*, *AT4G21070/BRCA1*, *At5g20850/RAD51*, and *AT4G37490/CYCLINB1;1* was not supported by the present transcriptome analysis of *hlq*, as none of these genes was altered (see Supplementary Datafile 1). Conclusions drawn from a meta-analysis of different microarray data sets are not absolute. It cannot be ruled out that genotype-, tissue-, or methodology-specific differences between the present experiments and those of others contributed to lack of agreement at the transcriptome level. However, it is also noted that contradictory results have been reported for the role of BIN4/MID in transcriptional silencing of heterochromatin (Breuer *et al.*, 2007; Kirik *et al.*, 2007). Since BIN4/MID does not physically interact with TOP6B/HLQ (Breuer *et al.*, 2007), the lack of similar molecular phenotypes for DNA damage markers in *hlq* mutants suggests that alternative mechanisms exist for regulation of TopoVI activities independent of BIN4/MID.

In this context, it can be recalled that *hlq* mutants were isolated on the basis of ectopic expression of the ABA- and auxin-inducible *proDc3:GUS* reporter gene in the epidermal cells of roots and hypocotyls (Subramanian *et al.*, 2002). Previous results showed that only a subset of *hlq* root epidermal cells stained strongly with the viable stain fluorescein diacetate (FDA). Remarkably *proDc3:GUS* staining exactly coincided with the FDA staining patterns, suggesting that FDA-marked epidermal cells specifically express *proDc3:GUS* and that these cells are metabolically hyperactive. The brassinolide insensitivity, skotomorphogenesis/hypocotyl elongation defect, and root hairless phenotypes of the *hlq* mutant were previously described (Subramanian *et al.*, 2002), consistent with the descriptions of other independent mutant allele phenotypes. The fundamental question is thus framed as ‘chicken versus egg’: is the *hlq/bin3/hyp6/rhl3/top6B* mutation causal for the expression of *proDc3:GUS*, which would thus serve as a *bona fide* marker for Top6B function in gene regulation, or rather is *proDc3*:*GUS* expression a downstream consequence of indirect internal stresses imposed by cell viability, wall defects/callose synthesis, or other secondary processes disrupted in the *hlq* mutant? The present results showing altered biosynthesis of primary metabolites such as starch (Figs 2, 6; Table 2) and cell walls (Figs 3, 7–9) in *hlq*, as well as *proDc3:GUS* expression in cell wall mutants of different classes (Supplementary Fig. S2 at *JXB* online) and following inhibition of peptidoglycan and cellulose biosynthesis (Supplementary Fig. S3) are consistent with a primary (direct) effect of *hlq* on gene expression. It is also possible that the lignin accumulation phenotype (Fig. 3) could cause alterations in cell–cell communication and thereby disturb hormonal signals or sensitivities.

The present meta-analysis of other transcriptome results showing the concordance of *bin5/top6a* and *caa39/top6a* with the *hlq/top6b* results further supports the chromatin remodelling model, because numerous differentially expressed unrelated adjacent genes could be identified (Supplementary Datafile 1 at *JXB* online) such as *At4g37250/LRR-NBS receptor kinase* and *At4g37260/MYB73*; *At5g23340/F-box* and *At5g23350/GRAM domain ABA responsive*; *At1g24140/matrixin metallopeptidase* and *At1g24150/formin homologue4*; *At1g28370/ERF-AP2 domain11* and *At1g28380/necrotic spotted lesions1*; *At1g66080*/unknown *and At1g66090/LRR-NBS receptor kinase*; *At1g74930/DREB subfamily A-5* and *At1g74940/DUF581*; *At2g18690*/unknown and *At2g18700/TSP11*; *AT3G25600/ EF-hand family protein* and *AT3G25610/haloacid dehalogenase*; *AT4G27652* and *AT4G27654* (unrelated unknowns); *At4g33040/thioredoxin* and *At4g33050/embryo sac defective39*; and *At5g13180/NAC083* and *At5g13190/LITAF domain.* All these gene pairs were coordinately regulated, supporting the generalized observation that *hlq/top6b* can impact chromatin remodelling, leading to transcription of unrelated adjacent genes. As previously noted (Simkova *et al.*, 2012) in the context of *top6a/caa39* characterization, there is *in vivo* molecular evidence that human TopoIIβ can generate transient double-stranded DNA breaks that permit exchanges in nucleosome-specific histone H1-HMGB proteins, which promotes local changes of chromatin structure and leads to the transcriptional activation of target genes (Ju *et al.*, 2006). Topoisomerase-mediated chromatin factor exchange represents an attractive mechanism for the transcriptional activation and repression of different sets of genes. More broadly, the present findings also raise questions about the forces exerted by chromatin remodelling factors in shaping plant genomes during evolution, for example if TopoVI was acquired by lateral gene transfer from archaebacteria instead of having been lost from the animal and fungal kingdoms. The finding of statistically significant misregulation of histone genes in the *hlq* mutant (Supplementary Fig. S13, Datafile S1, Table S4 at *JXB* online) including *At1g06760/HISTONE H1* is consistent with a chromatin factor exchange model and implies that there may be a feedback mechanism for TopoVI to regulate partner histone expression. The present systems approach of transcriptome meta-analysis revealed that *hlq* mutants misregulate a subset of ontologically unrelated genes based on proximity, which may help to explain the pleiotropic phenotypes of *bin3/hyp6/rhl3/top6b/hlq.*

Remarkably, mutants and inhibitors affecting the cell wall phenocopy several of the traits displayed in *hlq*. Plant cell walls are complex carbohydrate polymers, and it is estimated that ~10% of plant genomes are devoted to cell wall biogenesis. However, knowledge of their biogenesis and functions in signal transduction of environmental stimuli and plant development is limited, even in model organisms. Starch is the major non-structural carbohydrate in plants. It serves as an important store of carbon that fuels plant metabolism and growth when they are unable to photosynthesize. *Arabidopsis* has proven to be a powerful genetic system for discovering how starch is synthesized and degraded (Streb and Zeeman, 2012), and the present results (Figs 2, 6; Supplementary Fig. S9 at *JXB* online) suggest that *HLQ/TOP6B* can be added to the list of genes important for regulating starch and cell wall biosynthesis. However, caution should be exercised when inferring regulatory mechanisms because studies at the transcriptome level do not address post-translational regulation of metabolic pathways. Changes in sugar levels or other metabolic intermediates might serve as the trigger for the fluxes into and out of the starch pool. It was proposed that macro-autophagy may play a role in degrading non-functional chloroplasts in mutants of starch metabolism (Stettler *et al.*, 2009). Hartung *et al.* (2002) also observed, at the ultrastructural level in the cytoplasm of *top6b/spo11-2* and *top6a/atspo11-3* mutants, agglomerations of protein bodies, peroxisomes, and small vacuoles indicative of autophagy. There is genetic evidence that accumulation of starch degradation intermediates, such as disaccharides, causes severe cellular phenotypes such as chlorosis and chloroplast lysis, because mutations in *β-amylase2* and *β-amylase3*, which are strongly down-regulated in *hlq*, prevent the release of maltose from the starch granule and decrease the severe cellular chlorosis observed in the *maltose exporter1/dpe1* double mutant (Stettler *et al.*, 2009). Upon export to the cytosol, maltose is metabolized by a glucosyl transfer reaction catalysed by the cytosolic disproportionating enzyme At2g40840/DPE2. Mutants of *dpe2* accumulate starch and 200 times more maltose than the wild type, which causes severe chlorosis (Chia *et al.*, 2004; Stettler *et al.*, 2009), similar to the observed *hlq* gene expression phenotypes including significantly reduced *DPE2* expression (*P*=0.009; Supplementary Datafile 1), and elevated expression of several autophagy genes (Supplementary Fig. S11). The observation of significantly elevated expression of the two Glc6P/P_i_ transporter genes (*At5g54800/GPT1* and *At1g61800/GPT2*, *P*<0.003; Supplementary Datafile 1) is consistent with a model of starch metabolism intermediates functioning as regulators of carbon partitioning. Transcriptome profiling of *Arabidopsis* sucrose-inducible gene expression after 30min relief from starvation also revealed similar affected processes to those reported here: up-regulation of *GPT2* and differential expression of 21 transcription regulators (43% concordance), 15 ubiquitin-targeting proteins (60% concordance, but opposite FC), trehalose phosphate synthases (50% concordance, but opposite FC), autophagy protein 8e (opposite FC), several glutaredoxins (75% concordance, plus one *At1g28480/GRX480*, *P*<0.001), and carbon-scavenging enzymes for proline and myoinositol (all opposite FC: *At4g39800/Inositol-3-phosphate synthase/IPS1*; *At3g30775/proline oxidase*; and *myoinositol oxygenases MIOX2/4/At2g19800/At4g26260*) (Osuna *et al.*, 2007). That study also reported strong repression of *At2g17880/DnaJ* and of lipid catabolism genes after sucrose addition that mirrored the decrease of fatty acids in carbon-starved seedlings and their gradual recovery after sucrose addition. Of the handful of genes in Table 3 identified from meta-analysis as highly significant, one is a down-regulated lipid biosynthetic gene and another is *At2g17880/DnaJ*. Furthermore, the pervasive down-regulation of over-represented GDSL-motif lipases (*P*=0.01; Supplementary Fig. S12; Supplementary Datafile 1) suggests that lipids are being oxidized to fuel gluconeogenesis. These correlations strongly support the interpretation that *hlq* seedlings are starving, especially when taken together with results (Fig. 6, Table 2) that show that *hlq* leaves accumulate starch in the dark. Alternatively, the observed strong statistical association of *hlq* differentially expressed genes with protein degradation (Supplementary Fig. S10) is consistent with the phenotypes of the *det/cop/fus* class of mutants disrupting the COP9 signalosome, which is structurally and functionally similar to the 19S regulatory particle of the 26S proteosome and interacts with SCF-type E3 ubiquitin ligases to regulate targeted protein degradation (Smalle and Vierstra, 2004; Gusmaroli *et al.*, 2007).

AGP2, the regulatory subunit of the rate-limiting starch biosynthetic enzyme, is inactivated under oxidizing conditions (Streb and Zeeman, 2012), a state that the present transcriptome data and previous results (Simkova *et al.*, 2012) indicate exists in *hlq/top6b* and *top6a/caa39* mutants. Soluble starch synthase1 (At5g24300/SS1) has also been proposed to be regulated by the redox state (Glaring *et al.*, 2012), and it was observed here to be up- regulated (*P*=0.005; Supplementary Datafile 1 at *JXB* online) in *hlq*, which is an incongruity compared with other starch biosynthesis genes. In potato, high levels of glucose and sucrose increase both the AGP activation state and starch synthesis. The signalling processes for AGP regulation have been proposed to occur through hexokinase for glucose and through SnRK1 kinases for sucrose (Tiessen *et al.*, 2003). *HEXOKINASE1/GLUCOSE-INSENSITIVE2* was slightly up-regulated in *hlq* (*P*<0.05; Supplementary Datafile S1), whereas SnRK1 kinases were not affected. However, several of the 35 SnRK2/3 genes were significantly up-regulated (*P*<0.04; SnRK2.3; SnRK3.14/CIPK6/SOS3-INTERACTING3; SnRK3.10/CIPK7; SnRK3.15/CIPK14/PSK24; Supplementary Datafile S1) or down-regulated (*P*<0.008; SnRK2.1/ASK2; SnRK3.12/CIPK9/PKS6; SnRK3.17/CIPK3). Interestingly, these particular SnRKs have been shown to function in ABA, osmotic, and salt stress, flavonoid/sugar and calcium signalling, and auxin responses (Luan *et al.*, 2002; Kolukisaoglu *et al.*, 2004; Luan, 2009; Tripathi *et al.*, 2009; Heyndrickx and Vandepoele, 2012), all processes described herein as affected by the *hlq* mutation and manifest in the pleiotropic phenotypes. Likewise, the *hlq* phenotypes of abnormal root hair development, sterility, and biotic stress responses through LRR-NBS receptor kinases is known to be controlled by calcium and ROS (Monshausen *et al.*, 2008, 2009), processes significantly altered in *hlq* (Supplementary Fig. S11; Supplementary Datafile S1). Furthermore, there is evidence that the disaccharide trehalose-6-phosphate (Tre6P) acts as a signalling intermediate in the sucrose-dependent activation of AGP (Lunn *et al.*, 2006), which is strongly down-regulated in *hlq* (Supplementary Datafile S1). The finding that the trehalose marker *PAD4* and Tre6P biosynthetic gene *TREHALOSE PHOSPHATE SYNTHASE11* were strongly up-regulated in *hlq* supports this model. However, the details of this mechanism remain to be elucidated, and the unexpected down-regulated expression of the *AGP2* gene in *hlq* is not understood. Further analyses of trehalose and Tre6P in *hlq* and hypomorphic alleles could reveal the connections between the pathways of starch metabolism and the signalling pathways that regulate them, and provide insights into cell wall integrity-related mechanisms underlying environmental sensing, cell expansion, and morphogenesis (Hématy *et al.*, 2007; Boisson-Dernier *et al.*, 2011; Cheung and Wu, 2011).

The observed 1.9-fold up-regulation (*P*<0.0003) of *At2g03770/ST1-Like sulfotransferase*, involved in brassinosteroid metabolism (Marsolais *et al.*, 2007), is noted in connection with *bin3/hlq* skotomorphogenesis phenotypes. Similarly, the *hlq* mutant showed significant down-regulation (*P*<0.02) of a brassinosteroid biosynthetic gene *At5g05690/constitutive photomorphogenic dwarf/cabbage3/cyp90a1.* Mutants of *cpd/cbb3/cyp90a1* display de-etiolation and de-repression of light-induced genes in the dark, dwarfism, male sterility, and activation of stress-regulated genes in the light. Likewise, the observed strong down-regulation in *hlq* (Table 5) of *ABA1*, *CLA1*, *CED1*, and *NCED1* involved in ABA biosynthesis, skotomorphogenesis via cartenoid metabolism, and regulation of cell elongation and morphogenesis (Barrero *et al.*, 2008) is intriguing. Like *hlq*, mutants of *ced1/bodyguard/alpha-beta hydrolase* are dwarfed, extremely sensitive to osmotic stress, and have abnormal leaves, collapsed cells, and reduced numbers of trichomes (Wang *et al.*, 2011). Taken together with the observed misregulation of lipid and isoprenoid metabolism pathways in *hlq* (Supplementary Fig. S9, Supplementary Datafile S1), it is speculated that TopoVI regulation of genes controlling wax or lipid metabolism may be the molecular mechanism underlying the unexpected genetic link (Wang *et al.*, 2011) between cuticle biogenesis and ABA and osmotic stress responses. It is also speculated that TopoVI regulation of *AT1G01550/BYPASS1* and *At4g01360/BYPASS3* (significantly up-regulated in *hlq*; *P*<0.001, Supplementary Datafile S1) underlies the pleiotropic phenotypes of *bps1/3* mutants affecting root production of a mobile shoot morphogen of unknown structure derived from carotenoids (Lee *et al.*, 2012).

The findings that *hlq*/*top6b* mutants resemble the *det/cop/fus* class of mutants which accumulate the secondary metabolites anthocyanins parallels several recent lines of genetic evidence that responses to environmental cues are tightly coupled through sensors that regulate carbon partitioning between primary (aromatic amino acid) and secondary (polyphenolic) metabolites (Tzin and Galili, 2010). In addition to being a primary metabolite for protein synthesis, phenylalanine availability is rate limiting for production of phenylpropanoids important for physiology, UV protectants, fragrances/flavours related to reproduction, defence, hormonal pathways, and the synthesis of cell wall material that can comprise up to 50% of captured photosynthetic carbon, depending on the species. One possible insight into the cell elongation, lignin and anthocyanin accumulation phenotypes of *hlq* (Fig. 3A, B; Supplementary Figs S1C, S5D at *JXB* online) is that stem elongation, biomass, and lignin biosynthesis in vascular bundles and fibre cells are controlled by stem- and root-specific expression of chloroplastic arogenate dehydratases *At3g44720/ADT4* and *At5g22630/ADT5* (Corea *et al.*, 2012), which were significantly up-regulated in *hlq* mutants (*P*<0.0007; Supplementary Datafile 1). Another interesting facet is the link between ethylene and auxin signalling and transport, root hair development, cell elongation, and flavonols (Lewis *et al.*, 2011): *At2g20610/SUPERROOT1/HOOKLESS3/ROOTY* is an auxin overexpressor encoding an aminotransferase that functions in cross-talk between auxin and ethylene and is a marker for glucosinolate signalling (Clay *et al.*, 2009), and it was significantly down-regulated in *hlq* (*P*<0.01; Supplementary Datafile 1). Remarkably *dc3 overexpressor2* (*dor2*) was isolated and shown that it was a new allele of *sur1/rty* from the same screen that produced *hlq* (Sun, 2003). Further analysis and classification of up- and down-regulated genes in other TopoVI subunit mutants and characterization at the genetic, protein, and molecular levels of a series of hypomorphic TopoVI subunit B alleles (Fig. 5) may reveal the core function(s) of TopoVI. Studies on regulation of phenylalanine and polyphenolic biosynthetic pathways in both the chloroplast and cytosol, including plasma membrane-localized processes, should help unravel the complexities of lignin, cellulose, and polyphenolic contributions to structural integrity and cellular functions at the transcriptional, metabolic, and tissue levels.

## Supplementary data

Supplementary data are available at JXB online.

Figure S1. Pleiotropic phenotypes of the *hlq* mutant.

Figure S2. Ectopic *proDc3:GUS* expression and lignification in two cell-wall related mutants *procuste1-1* and *botero1-1*.

Figure S3. Cell wall inhibitors tunicamycin and DCB phenocopy epidermal traits of *hlq.*

Figure S4. Sanger sequencing results on both strands of independent amplicons spanning the *hlq* point mutation at chr3: 7267232, confirming the Illumina whole-genome re-sequencing result of G→A transition.

Figure S5. Dwarf, chlorotic, and anthocyanin-accumulation phenotypes of homozygotes for T-DNA insertion lines in *At3g20780* and that disrupt exons 4 and 12.

Figure S6. Pleiotropic root hair defects of anisotropic growth, bulging, and branching for T-DNA insertion lines in *At3g20780*/*TOP6B*.

Figure S7. Non-complementation (segregating mutants) in F_1_ progeny of crosses between heterozygous *hlq/+* and heterozygous T-DNA insertion lines, validated by PCR.

Figure S8. Volcano plot of the *hlq* transcriptome profiling experiment, showing the relationship between fold change effects of the *hlq* genotype compared with the wild type and statistical significance based on three replicates for ~25 000 genes on the microarray.

Figure S9. Transcriptome profiling of *hlq* seedlings for general metabolic pathway effects using MAPMAN (Usadel *et al.*, 2005).

Figure S10. Transcriptome profiling of *hlq* seedlings for ubiquitin- and autophagy- dependent protein degradation pathways using MAPMAN.

Figure S11. Transcriptome profiling of *hlq* seedlings to ‘Gene Regulation Overview’ using MAPMAN.

Figure S12. Transcriptome profiling of *hlq* seedlings to (A) ‘Cellular Response Overview’ and (B) ‘Large Enzyme Families’ pathways using MAPMAN.

Figure S13. Transcriptome profiling of *hlq* seedlings to specific families of transcription factors using MAPMAN.

Datafile S1. Transcriptome Microarray log_2_FC of *hlq*/WT, with statistical significance for differential expression (sheet 1). Sheet 2: overview of statistically significant over-represented processes (Benjamini–Hochberg corrected) affected by *hlq*, based on Wilcoxon Rank Sum Test of the transcriptome data set calculated by MAPMAN. Sheet 3: list of genes in statistically over-represented bins.

Data file S2. Detailed characterization of the *hlq* transcriptome.

Table S1. Cell length parameters of wild-type and *hlq* mutant hypocotyl and roots.

Table S2 (Datafile 1, sheet 4). Analysis of the 1079 most differentially expressed genes in the *hlq* mutant for chromosomal adjacency and co-regulation, compared with a comparable number of ‘control’ genes with the smallest FC symmetrically distributed about FC=0.

Table S3 (Datafile 1, sheet 5). Concordance between *hlq* transcriptome results and previously published lists of mis-regulated genes in *bin3/top6b* (Yin *et al.*, 2002) and *top6a/caa39* (Simkova *et al.*, 2012).

Table S4. List of HISTONE, WRKY, and AUX/IAA genes significantly mis-regulated in *hlq* mutant.

Table S5 (Datafile 1, sheet 6). Analysis of concordance between *hlq* misregulated genes compared with published transcriptome data sets for ABA RESPONSI VE17-(PYRABACTIN-RESISTANCE-LIKE12 homologous) overexpressing transgenic plants (Krishnaswamy *et al*., 2008) and ABA-regulated genes (Matsui *et al*., 2008).

Table S6. Primers for new markers developed during fine-mapping the *hlq* mutant, and other primers.

Supplementary Data
