# Supplementary material for: TOPOISOMERASE 6B is involved in chromatin remodelling associated with control of carbon partitioning into secondary metabolites and cell walls, and epidermal morphogenesis in Arabidopsis
Source: J Exp Bot. 2014 May 12;65(15):4217–39. doi: 10.1093/jxb/eru198 (PMC4112631; doi:10.1093/jxb/eru198)
Supplement: Supplementary Data [file supp_65_15_4217__index.html]

 TOPOISOMERASE 6B is involved in chromatin remodelling associated with control of carbon partitioning into secondary metabolites and cell walls, and epidermal morphogenesis in Arabidopsis — Supplementary Data 

# *TOPOISOMERASE 6B* is involved in chromatin remodelling associated with control of carbon partitioning into secondary metabolites and cell walls, and epidermal morphogenesis in *Arabidopsis*

## Supplementary Data

Data files

**Files in this Data Supplement:**

- Supplementary Data - Supplementary Data
- Supplementary Data - Supplementary Data
