# Supplementary material for: TOPOISOMERASE 6B is involved in chromatin remodelling associated with control of carbon partitioning into secondary metabolites and cell walls, and epidermal morphogenesis in Arabidopsis
Source: J Exp Bot. 2014 May 12;65(15):4217–39. doi: 10.1093/jxb/eru198 (PMC4112631; doi:10.1093/jxb/eru198)
Supplement: Supplementary Data [file supp_eru198_Mittal_hlq_JXB_supplemental_text_figs_tables146_revisedfonts.pdf]

Mittal et al. **Supplemental Datafile 2. Detailed characterization of *hlq* transcriptome.**

Up-regulation of several genes for respiration/mitochondrial electron transport was observed in the *hlq* mutant (Suppl. Datafile 1), possibly a consequence of reduced tetrapyrrole/heme/phytochrome chromophore biosynthesis and indicative of energy starvation and an oxidizing state in cells (Vanlerberghe and McIntosh, 1997). Four carbonic anhydrase genes, important for diffusion of both $C0_2$ and $HC0_3^-$ across the chloroplast stroma (Badger and Price, 1994), were strongly down-regulated, likely due to either a reduced photosynthetic demand for, or greater abundance of, CO_2 in *hlq* mutants.

The strongly down-regulated starch biosynthesis genes *At5g19220/ADP-Glucose Pyrophosphorylase2 (AGP2*) regulatory subunit (-3.5 FC), *At5g46110/triose-phosphate translocator* (*TPT*; -2.22 FC), *At4g17090/β-amylase3* (-1.91 FC), and *At4g00490/ β-amylase2* (-1.78 FC) were inversely correlated with strongly elevated expression of several genes for the reverse-flux assimilate pathway of sucrose biosynthesis (*viz. At3g13790/β-fructofuranosidase/cell wall invertase*, 2.96 FC; *At4g02280/sucrose synthase3*, 1.92 FC) and *At4g10120/sucrose-phosphate synthase-4F*, 2.61 FC). Mutants of *AGP2* have low starch levels (Lin et al., 1988), and thus the observed repressed expression of *AGP2* in *hlq* is paradoxical since *hlq* mutants accumulate starch (Figs. 2, 6). However, it is known that mutants of *TPT* have a starch excess phenotype due to reduced export of the triose-phosphate from the chloroplast (Schneider et al., 2002), and mutants of the starch degradation genes *At3g52180/phosphoglucan phosphatase/SUCROSE EXCESS4, At2g40840/4-α-glucanotransferase disproportionating enzyme2/DPE2*, and *β-amylase3* accumulate starch (Kaplan and Guy, 2005; Kotting *et al.*, 2009; Lu and Sharkey, 2006), consistent with their observed down regulation ($p < 0.01$; Suppl. Datafile 1) in *hlq*. Trehelose (Tre) is α-1, 1-linked glucose disaccharide thought to function in regulation of sugar signaling and biotic stress. Experiments have shown exogenous Tre provided to Arabidopsis seedlings alters carbon allocation, with massive starch accumulation in cotyledons and leaves (Schluepmann and Paul, 2009), and induces *At3g52430/PHYTOALEXIN DEFICIENT4* (Singh et al., 2011). We observed very strong up-regulation (3.72 FC, $p < 0.00005$) of *At2g18700/TREHELOSE PHOSPHATE SYNTHASE11/TSP11*, and ~1.5-fold induction of *PAD4* and *At1g23870/TSP9* ($p < 0.006$)(Suppl. Datafile 1) which suggests these

genes may be signaling starch accumulation via Tre accumulation, and possibly secondary metabolite anthocyanin accumulation (Suppl. Figs. S1C, S5D) in *hlq* through sugar sensing (Luo et al., 2012; Shin et al., 2013; Solfanelli et al., 2006).

A Wilcoxon Rank Sum Test on 'bins' for metabolism gene classes was performed with MAPMAN (Usadel et al., 2005), to determine whether objects within one functional class behave differently from the remaining objects. Statistically significant differences (Benjamini-Hochberg- false-discovery corrected *p*-values) in the degree of differential expression were found for genes in the following processes: PS light reactions (10^{-42}), PS Calvin cycle (10^{-8}), mitochondrial electron transport /ATP synthesis (10^{-4}), tetrapyrrole synthesis (10^{-4}), photorespiration (10^{-4}), C1-metabolism (10^{-3}), fatty acid synthesis and elongation (10^{-3}), tryptophan and aromatic aa metabolism (0.005; all up-regulated), cytosolic glycolysis (0.009), and carotenoids/non-melavonate metabolism (0.006; all down-regulated).

We performed similar analyses of process classes 'Gene Regulation Overview', 'Cell Functions Overview', 'Cellular Response Overview', and 'Large Enzyme Families' pathways. Specific processes exhibiting statistically higher (*p*-value) differential gene expression in our *hlq* dataset were those involving protein degradation (10^{-9}), especially sub-bins ubiquitin-proteasome (10^{-8}), E3-ligase Skip-Cullin-F box complex (10^{-4}), E3-ligase RING components (p= 0.01), and autophagy (p = 0.08)(Suppl. Fig. S10). Also significantly affected were the processes of biotic stress (10^{-5}), protein targeting to chloroplast and mitochondria ($p < 0.02$), signaling (p <0.03), calcium signaling (p = 0.01, all up-regulated; Suppl. Fig. S11), GDSL-motif lipases (p = 0.01; all down-regulated; Suppl. Fig. S12), transport by ATPases ($p < 0.01$), major intrinsic proteins/aquaporins ($p < 0.002$), and RNA regulation of transcription ($p < 0.05$). Supplemental Figures S11- S13 show graphically the general bin category results; individual genes for each of these processes are listed in Supplemental Datafile 1. It is interesting to note a recent report links calcium signaling with sugar-induced anthocyanin biosynthesis (Shin et al., 2013), supporting our observed correlations (see above).

Down-regulation of photosynthetic genes and up-regulation of mitochondrial electron transport genes in *hlq* correlated with observed deficiencies in chlorophyll accumulation and elevated uptake/metabolism of the viability stain fluorescein diacetate (Subramanian et al., 2002). Taken together these observations suggested that in the absence of a properly functioning

photosynthetic apparatus, ROS may be elevated in *hlq/top6b*. This inference was recently validated by cloning and transcriptome characterization of *top6a/caa39* and characterization of marker gene expression in *hyp6/top6b* (Simkova et al., 2012). Supporting evidence for an abnormal oxidative state was the strong up- and down-regulation of glutathione and ascorbate oxido-reductases and peroxidases, and down-regulation of *At4g37930/SERINE TRANSHYDROXYMETHYLTRANSFERASE-1*, mapped on the metabolic grid (Suppl. Fig. S9; Suppl. Datafile 1) under C1 metabolism but also functioning in photorespiration and control of cell damage caused by abiotic stress (Moreno *et al.*, 2005; Voll *et al.*, 2006). It is intriguing that our *hlq* transcriptome results for ROS activities and ubiquitinylation/proteasome/autophagy pathways (see above, Suppl. Fig. S10) correlate with the finding that oxygen sensing is mediated by an N-end rule pathway (Licausi et al., 2011).

Germin and germin-like proteins have been shown to have superoxide dismutase activity (Christensen et al., 2004), and all members of this class except one were significantly mis-regulated in *hlq*, especially *At4g14630/GERMIN-LIKE PROTEIN9/GLP9*, *At1g72610/GLP1*, *At1g09560/GLP5*, *At5g39100/GLP6*, *At5g39190/GLP2A*, and *AT5G20630/GLP3* ($p < 0.001$; Suppl. Datafile 1). The strong up-regulation of numerous peroxidases observed by transcriptome microarrays (Suppl. Datafile 1) was confirmed by an in-gel enzymatic assay that showed up-regulation of three cell wall peroxidase isozymes in *hlq* (Suppl. Fig. S12C). Peroxidase activity in cell walls of plants is presumed to be involved with extensin and hydroxyproline-rich protein cross-linking, lignification, suberization, disease resistance and wound-healing (Almagro et al., 2009; Bindschedler et al., 2006; Choi et al., 2007; Minibayeva et al., 2009).

Based on our transcriptome results (Table 4) showing associations between mis-regulated genes in *hlq* and documented effectors of ABA responses, we analyzed for concordance with our results the top genes reported from two independent transcriptome datasets that interrogated ABA responses explicitly (Matsui et al., 2008) or implicitly (Krishnaswamy et al., 2008). The pea gene *ABA RESPONSE17/ABR17* when over-expressed in Arabidopsis and Brassica results in enhanced germination and seedling growth and elevated expression of ABA-responsive genes, especially in response to salt stress (Krishnaswamy et al., 2008). In the Arabidopsis genome, ABR17 (Iturriaga et al., 1994) is most homologous to ABA receptor PYRABACTIN-RESISTANT-LIKE12/RCAR6 (56% and 66% similarity to pea and Medicago proteins,

respectively), an aspect not explored to date in regards to ABA signaling per se. Supplemental Table S5 lists the top 200 genes reported as most up- and down-regulated by 10 h ABA treatment of Arabidopsis whole seedlings (Matsui et al., 2008), and analysis of concordance for > 1.5 FC up- or down-regulation in the *hlq* mutant ($p < 0.003$). For both classes of indicated genes (ABA up- and down-regulated), >70% of genes were regulated in *hlq* in the correct direction (up versus down). 31% of indicated genes were concordantly up-regulated and 26% concordantly down-regulated > 1.5-fold, respectively, in the *hlq* mutant, which is a good concordance on par or better than observed for the two analyzed transcriptome datasets for other mutant alleles of *top6a* and *top6b* (see above; Suppl. Table S3). For ABR17 over-expression transgenics treated with or without NaCl stress, 40% of the indicated genes were concordant for significant (average $p < 0.004$) up- or down-regulation in *hlq* mutants, and likewise 70% of the indicated genes were altered in the correct direction (up versus down) in *hlq* (Suppl. Table S5).

Because *hlq* encodes a null allele of TOP6B, activity of transcription factors and chromatin is hypothesized to be primarily affected. Statistical analysis by MAPMAN of transcription factor and chromatin-associated gene classes showed that histone (10^{-4}) and WRKY domain ($p < 0.002$) classes of transcription factors were significantly up-regulated in *hlq* mutants, whereas AUX/IAA transcription factor class was significantly ($p < 0.01$) down-regulated (Suppl. Fig. S13; Suppl. Table S4). Supplemental Datafile 1 lists the transcription factors and other gene classes and their expression levels in *hlq* that were significantly over-represented. The observed uniform down-regulation of *AUX/IAA* genes in *hlq* might be due to the fact that they are targets of the SCF-ubiquitin targeted protein degradation pathway (Gray et al., 2001), which was significantly up-regulated in *hlq* (Suppl. Fig. S10), or possibly related to some of them being involved in brassinosteroid-regulated growth (Nakamura et al., 2006).

At1g18570/HIGH INDOLIC GLUCOSINOLATE1/MYB51 is the major regulator of anti-pathogen glucosinolate biosynthesis by up-regulation of mono-oxygenases *At4g39950/CYP79B2*, *At4g31500/CYP83B1/ ALTERED TRYPTOPHAN REGULATION4,* and *AT5G05730/ANTHRANILATE SYNTHASE1* and *At1g74100/CORONOTINE-INDUCED7/SULFOTRANSFERASE5A* (Clay *et al.*, 2009; Gigolashvili *et al.*, 2007; Malitsky *et al.*, 2008); all five genes were up-regulated in *hlq* ($p < 0.002$; Suppl. Datafile 1), supporting the hypothesis that TopoVI regulates Trp homeostasis and glucosinolates through a transcriptional

hierarchy, but feedback regulation from altered heme biosynthesis or indole metabolites in *hlq* mutants cannot be ruled out.

Supplemental References

Almagro L, Gómez Ros LV, Belchi-Navarro S, Bru R, Ros Barceló A, Pedreño MA. 2009. Class III peroxidases in plant defence reactions. *Journal of Experimental Botany* **60**, 377-390.

Badger MR, Price GD. 1994. The role of carbonic anhydrase in photosynthesis. *Annual Review of Plant Physiology & Plant Molecular Biology* **45**, 369-392.

Bindschedler LV, Dewdney J, Blee KA, Stone JM, Asai T, Plotnikov J, Denoux C, Hayes T, Gerrish C, Davies DR, Ausubel FM, Bolwell GP. 2006. Peroxidase-dependent apoplastic oxidative burst in Arabidopsis required for pathogen resistance. *Plant Journal* **47**, 851-863.

Choi HW, Kim YJ, Lee SC, Hong JK, Hwang BK. 2007. Hydrogen peroxide generation by the pepper extracellular peroxidase $CaPO_2$ activates local and systemic cell death and defense response to bacterial pathogens. *Plant Physiology* **145**, 890-904

Christensen AB, Thordal-Christensen H, Zimmermann G, Gjetting T, LyngkjÃ¦r MF, Dudler R, Schweizer P. 2004. The Germin Like Protein GLP4 exhibits superoxide dismutase activity and is an important component of quantitative resistance in wheat and barley. *Molecular Plant-Microbe Interactions* **17**, 109-117.

Gigolashvili T, Berger B, Mock H-P, Müller C, Weisshaar B, Flügge U-I. 2007. The transcription factor HIG1/MYB51 regulates indolic glucosinolate biosynthesis in *Arabidopsis thaliana*. *Plant Journal* **50**, 886-901.

Gray WM, Kepinski S, Rouse D, Leyser O, Estelle M. 2001. Auxin regulates SCFTIR1-dependent degradation of AUX/IAA proteins. *Nature* **414**, 271-276.

Iturriaga EA, Leech MJ, Barratt DH, Wang TL. 1994. Two ABA-responsive proteins from pea (Pisum sativum L.) are closely related to intracellular pathogenesis-related proteins. *Plant Molecular Biology* **24**, 235-240.

Kaplan F, Guy CL. 2005. RNA interference of Arabidopsis *Beta-Amylase8* prevents maltose accumulation upon cold shock and increases sensitivity of PSII photochemical efficiency to freezing stress. *Plant Journal* **44**, 730-743.

Kotting O, Santelia D, Edner C, Eicke S, Marthaler T, Gentry MS, Comparot-Moss S, Chen J, Smith AM, Steup M, Ritte G, Zeeman SC. 2009. STARCH-EXCESS4 is a laforin-like phosphoglucan phosphatase required for starch degradation in *Arabidopsis thaliana*. *Plant Cell* **21**, 334-346.

Krishnaswamy S, Srivastava S, Mohammadi M, Rahman M, Deyholos M, Kav N. 2008. Transcriptional profiling of pea ABR17 mediated changes in gene expression in *Arabidopsis thaliana*. *BMC Plant Biology* **8**, 91.

Licausi F, Kosmacz M, Weits DA, Giuntoli B, Giorgi FM, Voesenek LACJ, Perata P, van Dongen JT. 2011. Oxygen sensing in plants is mediated by an N-end rule pathway for protein destabilization. *Nature* **479**, 419-422

Lin T-P, Caspar T, Somerville CR, Preiss J. 1988. A starch deficient mutant of *Arabidopsis thaliana* with low ADPglucose pyrophosphorylase activity lacks one of the two subunits of the enzyme. *Plant Physiology* **88**, 1175-1181.

Lu YAN, Sharkey TD. 2006. The importance of maltose in transitory starch breakdown. *Plant, Cell & Environment* **29**, 353-366.

Malitsky S, Blum E, Less H, Venger I, Elbaz M, Morin S, Eshed Y, Aharoni A. 2008. The transcript and metabolite networks affected by the two clades of Arabidopsis glucosinolate biosynthesis regulators. *Plant Physiology* **148**, 2021-2049.

Matsui A, Ishida J, Morosawa T, Mochizuki Y, Kaminuma E, Endo TA, Okamoto M, Nambara E, Nakajima M, Kawashima M, Satou M, Kim JM, Kobayashi N, Toyoda T, Shinozaki K, Seki M. 2008. Arabidopsis transcriptome analysis under drought, cold, high-salinity and ABA treatment conditions using a tiling array. *Plant & Cell Physiology* **49**, 1135-1149.

Minibayeva F, Kolesnikov O, Chasov A, Beckett RP, Lüthje S, Vylegzhanina N, Buck F, Böttger. 2009. Wound-induced apoplastic peroxidase activities: their roles in the production and detoxification of reactive oxygen species. *Plant, Cell & Environment* **32**, 497-508.

Moreno JI, Martín R, Castresana C. 2005. Arabidopsis SHMT1, a serine hydroxymethyltransferase that functions in the photorespiratory pathway influences resistance to biotic and abiotic stress. *Plant Journal* **41**, 451-463.

Nakamura A, Nakajima N, Goda H, Shimada Y, Hayashi K-i, Nozaki H, Asami T, Yoshida S, Fujioka S. 2006. Arabidopsis *Aux/IAA* genes are involved in brassinosteroid-mediated growth responses in a manner dependent on organ type. *Plant Journal* **45**, 193-205.

Schluepmann H, Paul M. 2009. Trehalose metabolites in Arabidopsis: elusive, active and central. *The Arabidopsis Book*, e0122. http://www.bioone.org/doi/full/0110.1199/tab.0122.

Schneider A, Häusler RE, Kolukisaoglu Ü, Kunze R, Van Der Graaff E, Schwacke R, Catoni E, Desimone M, Flügge U-I. 2002. An *Arabidopsis thaliana* knock-out mutant of the chloroplast triose phosphate/phosphate translocator is severely compromised only when starch synthesis, but not starch mobilisation is abolished. *Plant Journal* **32**, 685-699.

Singh V, Louis J, Ayre BG, Reese JC, Shah J. 2011. TREHALOSE PHOSPHATE SYNTHASE11-dependent trehalose metabolism promotes *Arabidopsis thaliana* defense against the phloem-feeding insect *Myzus persicae*. *Plant Journal* **67**, 94-104.

Vanlerberghe GC, McIntosh L. 1997. ALTERNATIVE OXIDASE: from gene to function. *Annual Review of Plant Physiology and Plant Molecular Biology* **48**, 703-734.

Voll LM, Jamai A, Renne P, Voll H, McClung CR, Weber APM. 2006. The photorespiratory Arabidopsis *shm1* mutant is deficient in SHM1. *Plant Physiology* **140**, 59-66.

Supplemental Figures

Title: “*TOPOISOMERASE 6B* is involved in chromatin remodeling associated with hormone and environmental control of carbon partitioning, secondary metabolite and cell wall synthesis, and epidermal morphogenesis in Arabidopsis”

Authors: Amandeep Mittal, Rajagopal Balasubramanian, Jin Cao, Prabhjeet Singh, Senthil Subramanian, Glenn Hicks, Eugene A. Nothnagel, Noureddine Abidi, Jaroslav Janda, David W. Galbraith, and Christopher D. Rock

Supplementary Figure S1

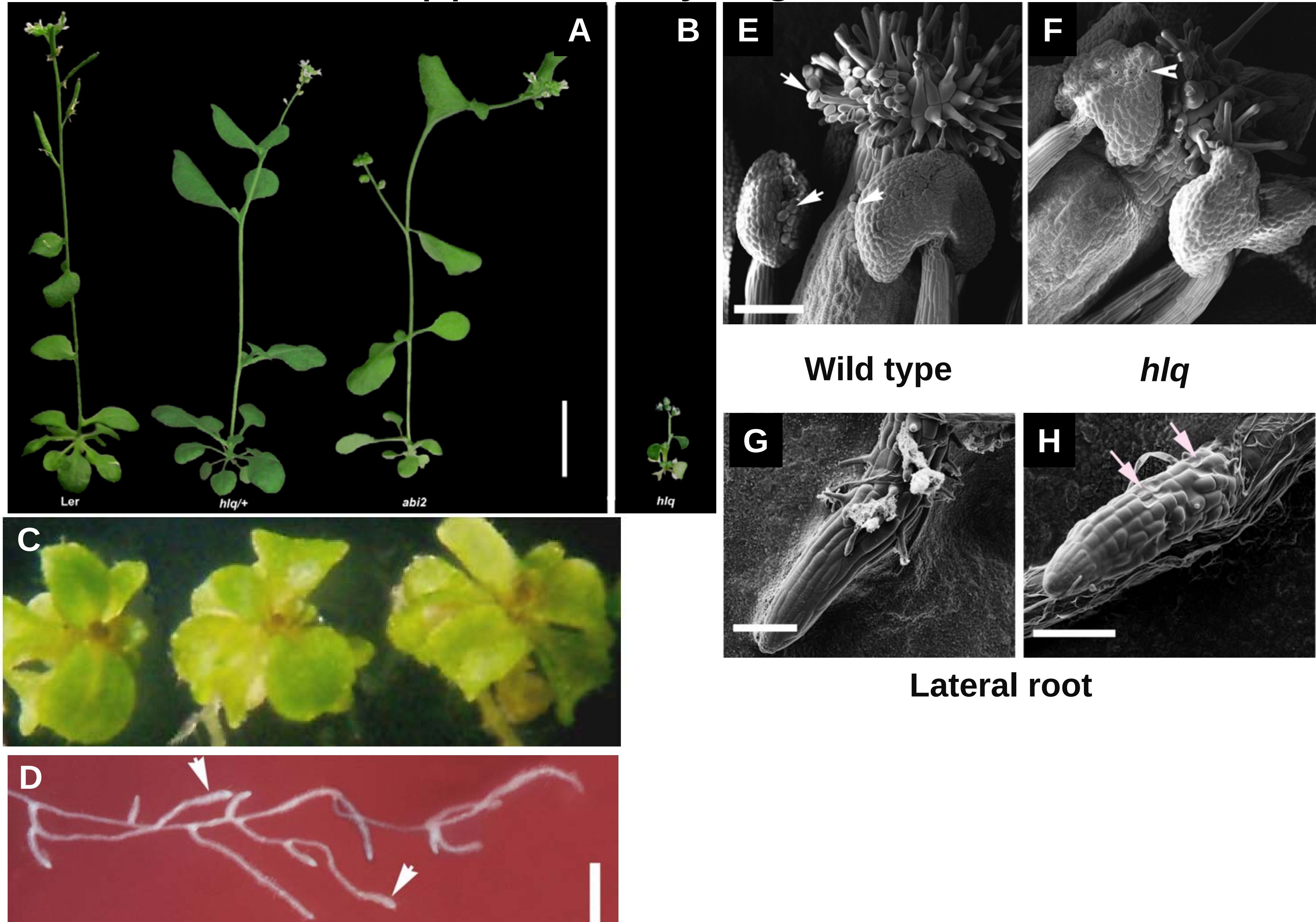

Suppl. Fig. S1 (above): Pleiotropic phenotypes of the *hlq* mutant. (A) Stature of three week-old wild-type and *hlq* plants. Stature of heterozygous *hlq* /+ is similar to that of the wild-type plants. All plants were grown in soil. (B) Stature of homozygous *hlq* grown in sucrose-containing media. Bar in (A) represents 2 cm for (A) and 1 cm for (B). (C) Two weeks old *Ler*/*hlq* seedlings (not in parent *abi2-1* background) grown on sucrose-containing media in light shows the rough surface of cotyledons, and accumulation of anthocyanins in the shoot apical meristem, reminiscent of *fusca* mutants. (D) Swollen root morphology of *hlq*. The root tips of *hlq* are swollen (arrows) when grown on minimal media. The phenotype is more pronounced after 2-3 weeks, similar to the cellulose-deficient *rsw* mutants. The bar represents 1 cm. (E, F) Scanning electron micrographs of wild-type (E) and *hlq* flowers (F). Micrographs were obtained from fully opened mature flowers. Note the dehiscence of wild-type anthers (E; solid arrows) with pollen germinating on the stigma. The stigma papillae are also uniform in size and shape. (F) The anthers of *hlq* do not dehisce and the rough adaxial surface of anther is pitted with clustered stomata (indented arrow). Note the uneven shape and size of the stigma papillae. Bar represents 10 µm. (G, H) Scanning electron micrographs of *hlq* and *abi2* parental lateral roots. Arrows show collapsed cells in *hlq* roots. Bar = 50 µm.

Supplementary Figure S2

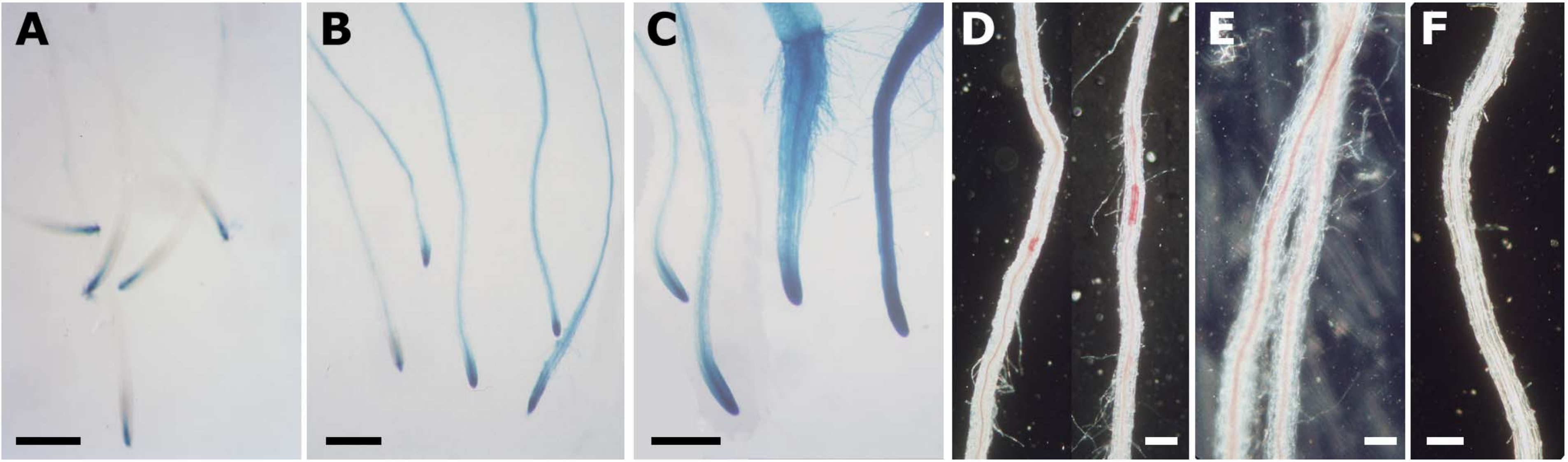

Fig. S2. Ectopic *Dc3-GUS* expression and lignification in two cell-wall related mutants *procuste1-1* and *botero1-1*. Constitutive *Dc3-GUS* expression in one-week old roots of (A) wild type, *prc1-1* (B) and *bot1-1* (C). Ectopic lignification detected by phloroglucinol in one week-old roots of *prc1-1* (D), *bot1-1* (E) compared to wild-type root (F) showing no staining. Scale bars, 500 µm in A-C; 100 µm in C-E.

Supplementary Figure S3

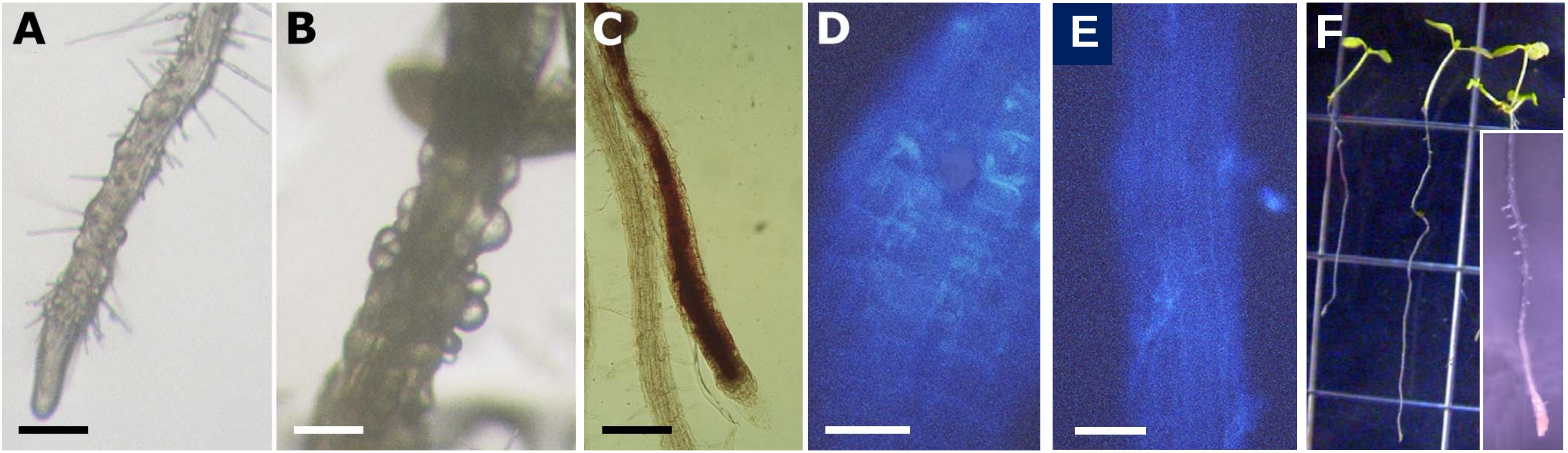

Fig. S3. Cell wall inhibitors tunicamycin and DCB phenocopy epidermal traits of *hlq*. Roots (A) and hypocotyls (B) of wild-type seedlings grown on tunicamycin (inhibitor of N-glycosylation involved in peptidoglycan synthesis) showed bulged cells. Ectopic lignification (C) and callose accumulation (D) was observed in the distal end of wild-type roots grown on tunicamycin, whereas the callose staining pattern in an untreated root (E) was very faint and limited to the intercellular regions. For (C), the root on the left shows the zone of differentiation; the root on the right is the primary root tip and zone of elongation. Scale bar = 100 µm, except E: 50 µm. (F) In the presence of 1 µM DCB (a cellulose-biosynthesis inhibitor), production of lateral roots and root hairs in wild type seedlings was inhibited and the root zone of elongation swelled (inset; close-up of the root-tip), phenocopying the *hlq* mutant.

Supplementary Figure S4

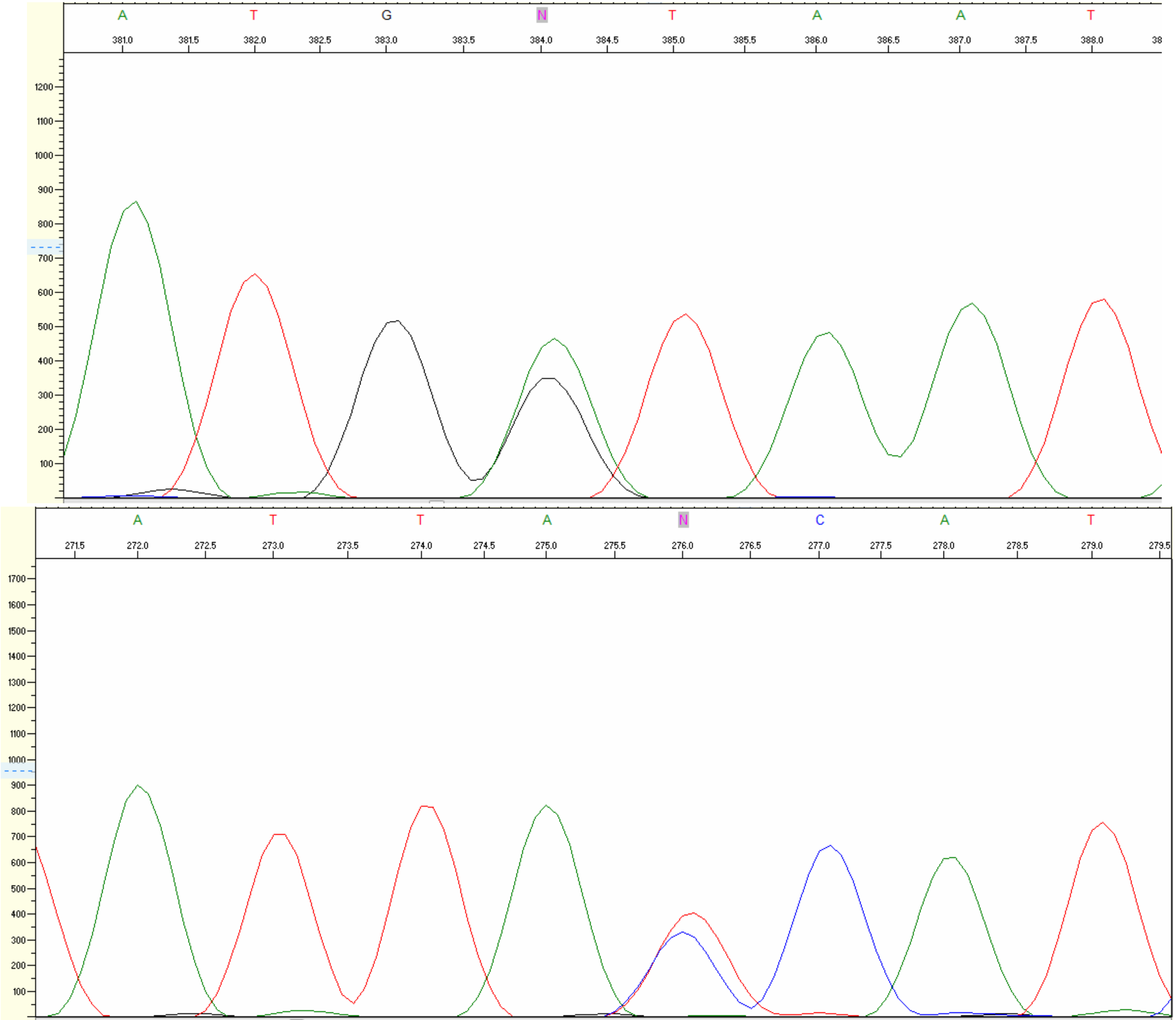

Fig. S4 (above): Sanger sequencing results on both strands of independent amplicons spanning the *hlq* point mutation at chr3:7267232, confirming the Illumina whole-genome re-sequencing result of G→A transition.

Fig. S5 (below): Dwarf, chlorotic, and anthocyanin-accumulation phenotypes of homozygotes for T-DNA insertion lines in *At3g20780* and that disrupt exons 4 and 12. A) Wild type Col-0, grown vertically on phytagel plates for 17 days. Black bar on left = 5 cm. B, C) 15 day old homozygous individuals of SALK_024455 and SALK_140704, respectively, showing chlorosis and anthocyanin accumulation. White bar in middle = 5 cm. D) Close up of SALK_024455 individual plantlet, showing anthocyanin accumulation in the meristem and leaf primordia. E) Size comparison of wild type seedling expanded leaf, and a T-DNA mutant plantlet.

Supplementary Fig. S5. Homozygous mutant plants from T-DNA insertion lines Salk_024455 and Salk_140704 are dwarf and chlorotic, like *hlq* mutants

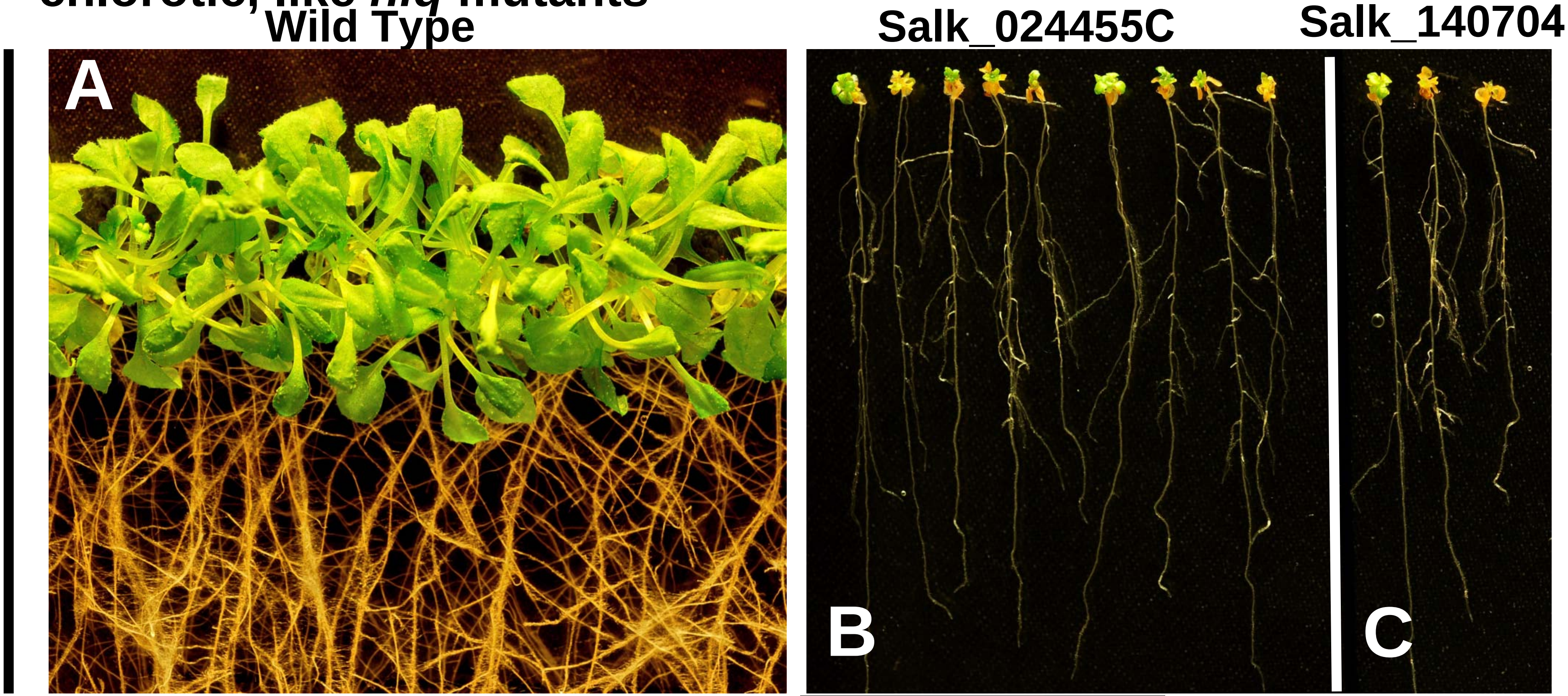

Panels A- C: Black (left) and white bars (right) are equal to 5 cm.

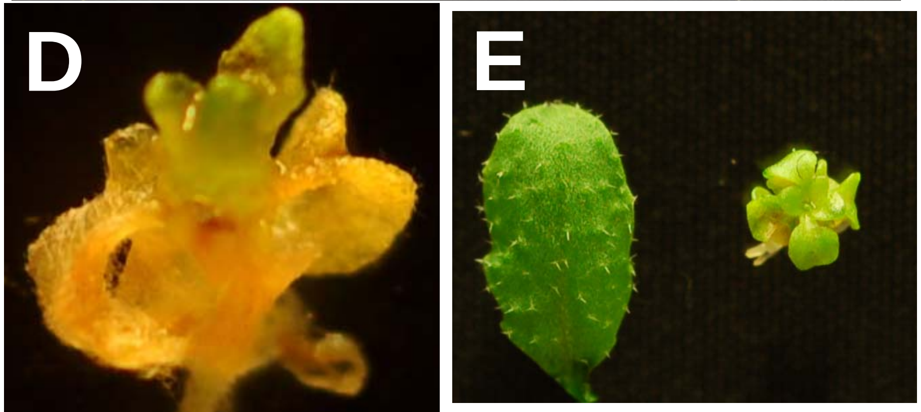

Supplementary Fig. S6. Pleiotropic root hair defects of anisotropic growth, bulging, and branching for T-DNA insertion lines in *At3g20780*/*TOP6B*. A) wild type Col-0 at 10X magnification. B) T-DNA insertion line SALK_140704 at 20X magnification. C) SALK_024455 at 20X magnification. D) SALK_024455 at 40X magnification. E) SALK_140704 branched root hair, imaged with a dissecting microscope.

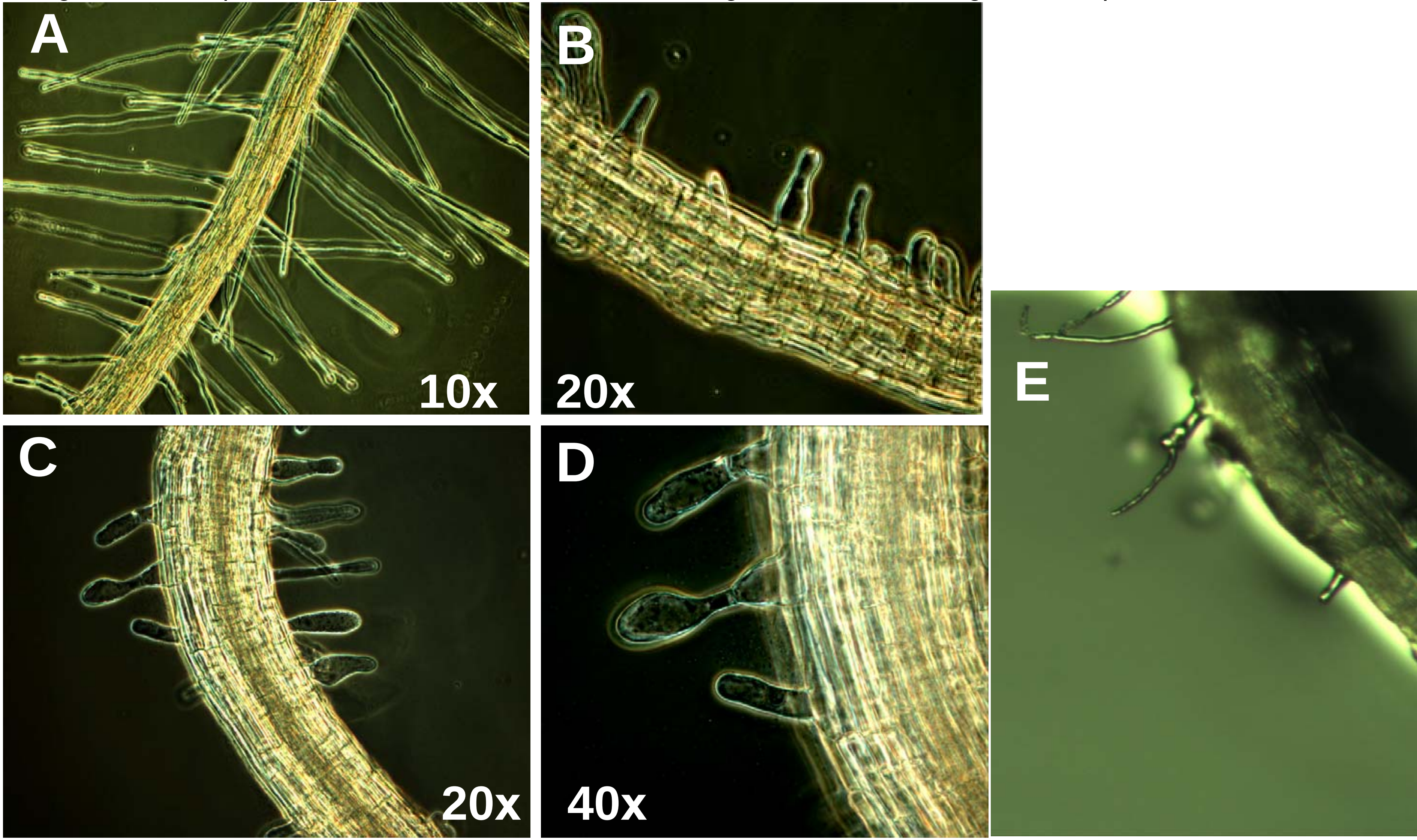

Supplementary Fig. S7. Non-complementation (segregating mutants) in F_1 progeny of crosses between heterozygous *hlq*/+ and heterozygous T-DNA insertion lines.

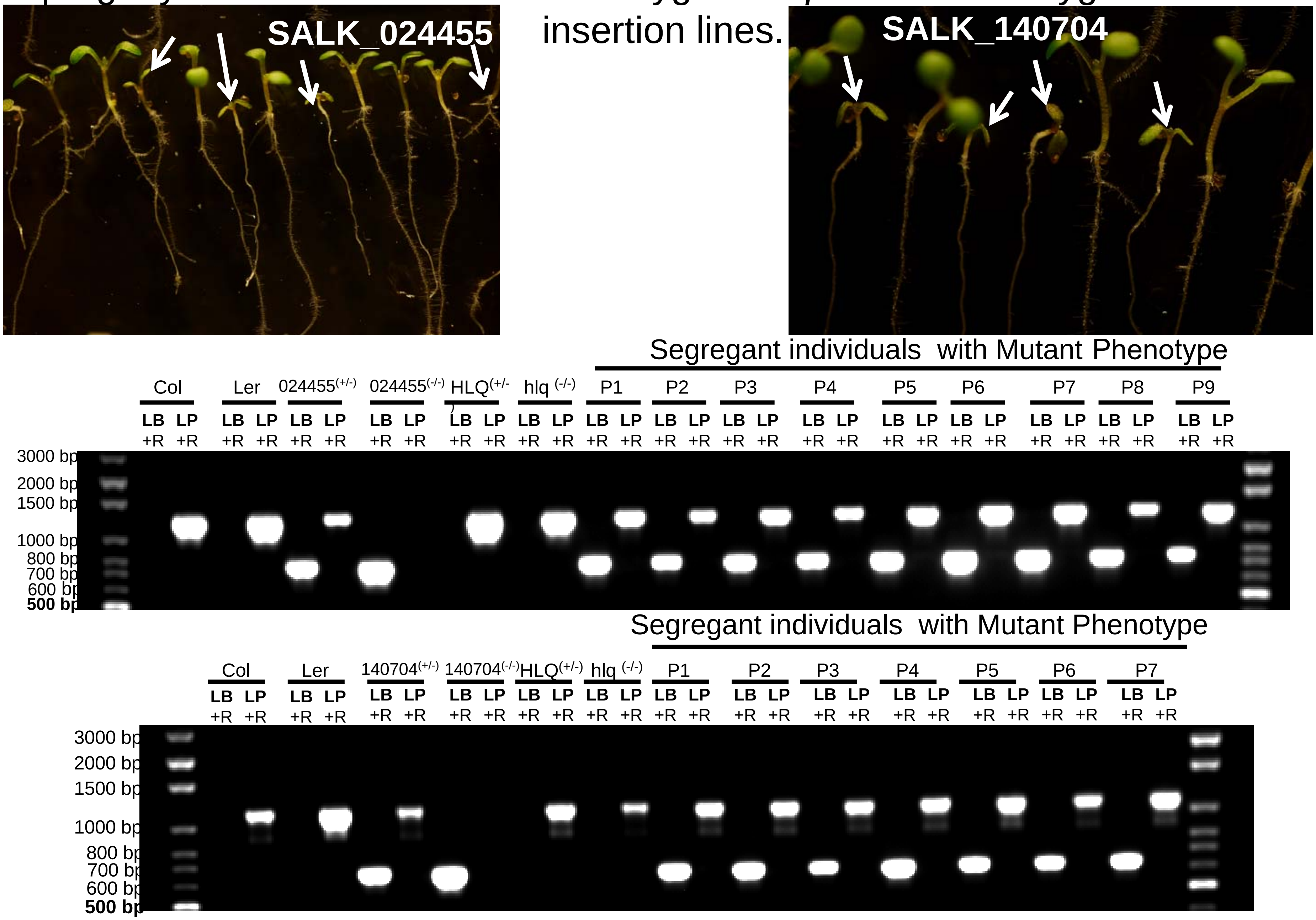

Supplementary Figure S8

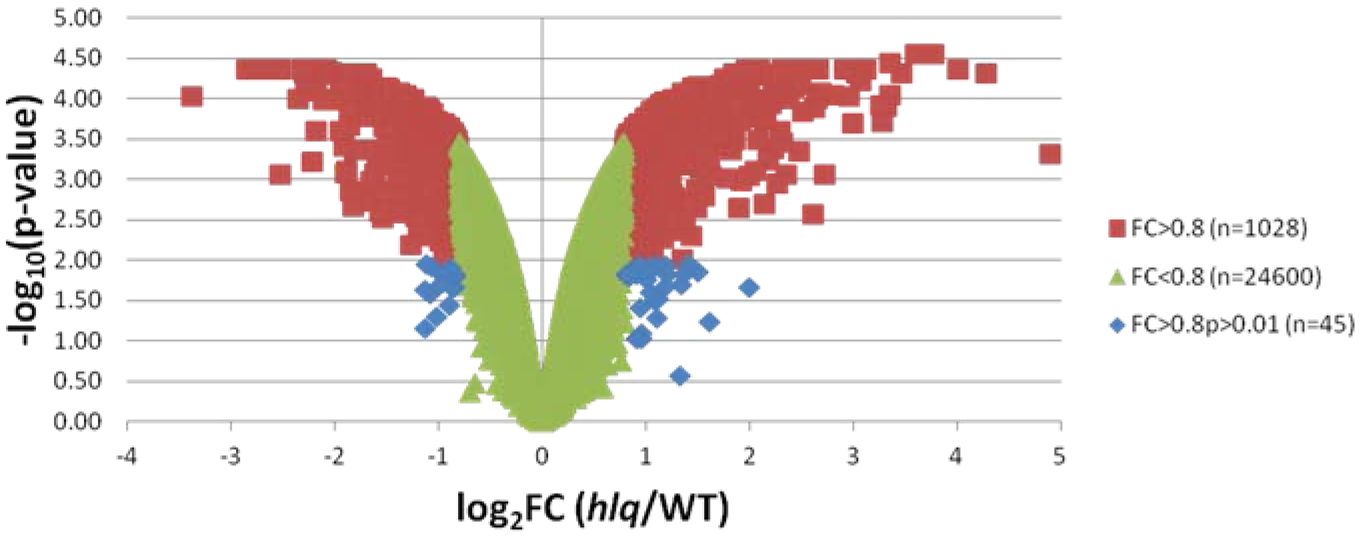

Fig. S8. Volcano plot of *hlq* transcriptome profiling experiment, showing relationship between fold-change effects of *hlq* genotype compared to wild type and statistical significance based on three replicates for ~25,000 genes on the microarray.

Fig. S9. Transcriptome profiling of *hlq* seedlings for general metabolic pathway effects using MAPMAN (Usadel et al., 2005).

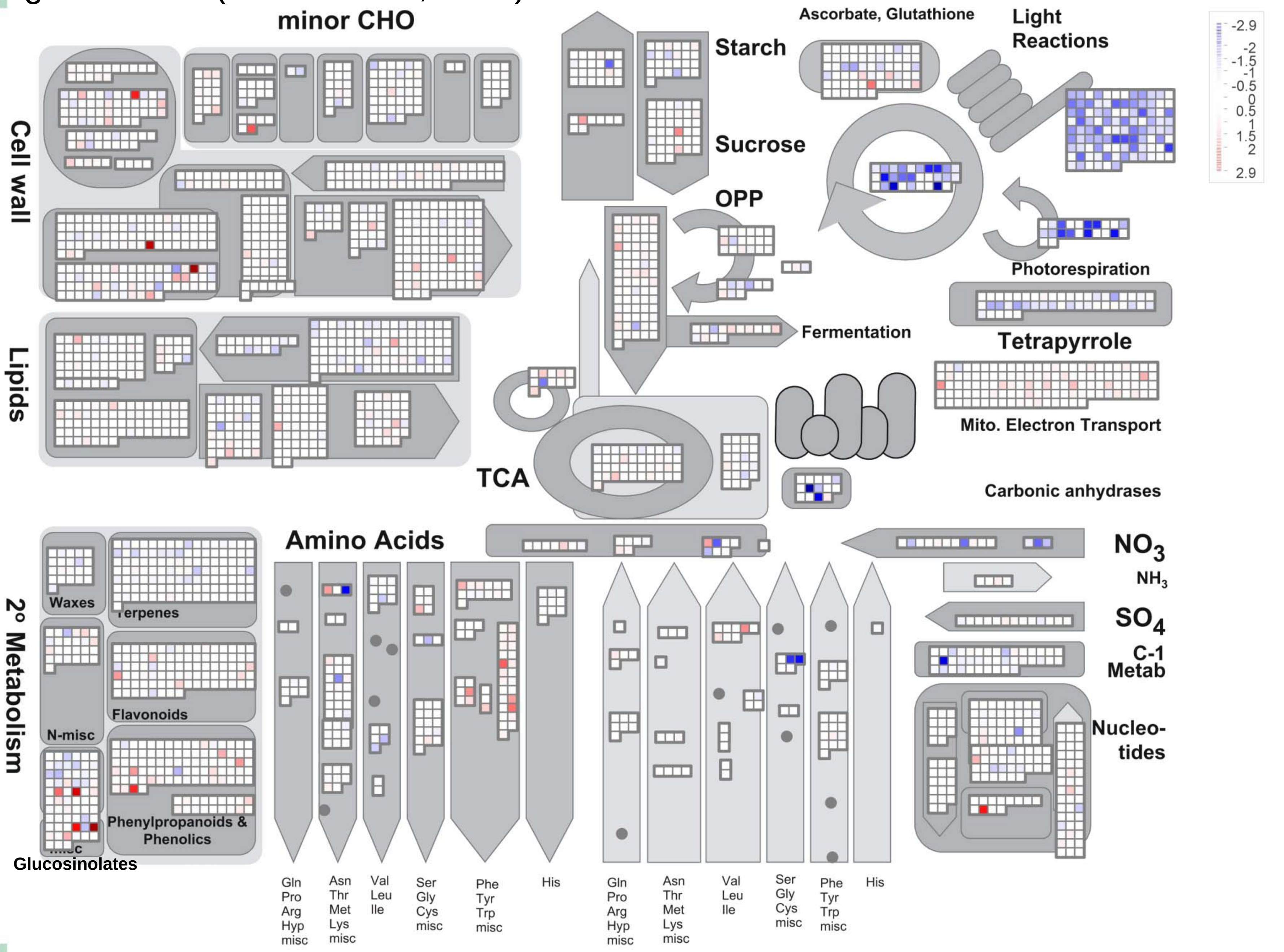

Supplementary Figure S10

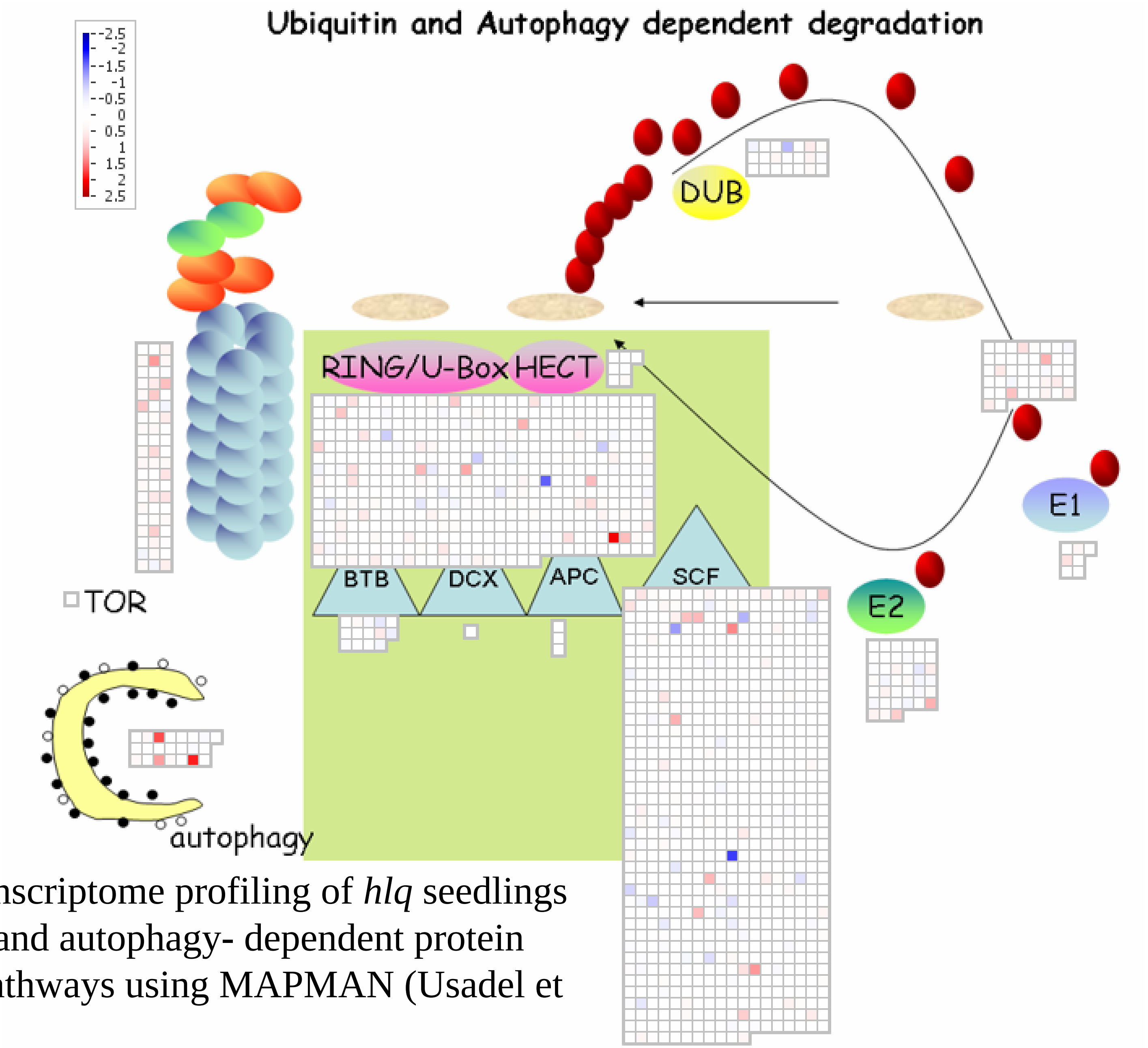

Fig. S10. Transcriptome profiling of *hlq* seedlings for ubiquitin- and autophagy- dependent protein degradation pathways using MAPMAN (Usadel et al., 2005).

Supplementary Figure S11: Transcriptome profiling of *hlq* seedlings to ‘Gene Regulation Overview’ using MAPMAN (Usadel et al., 2005).

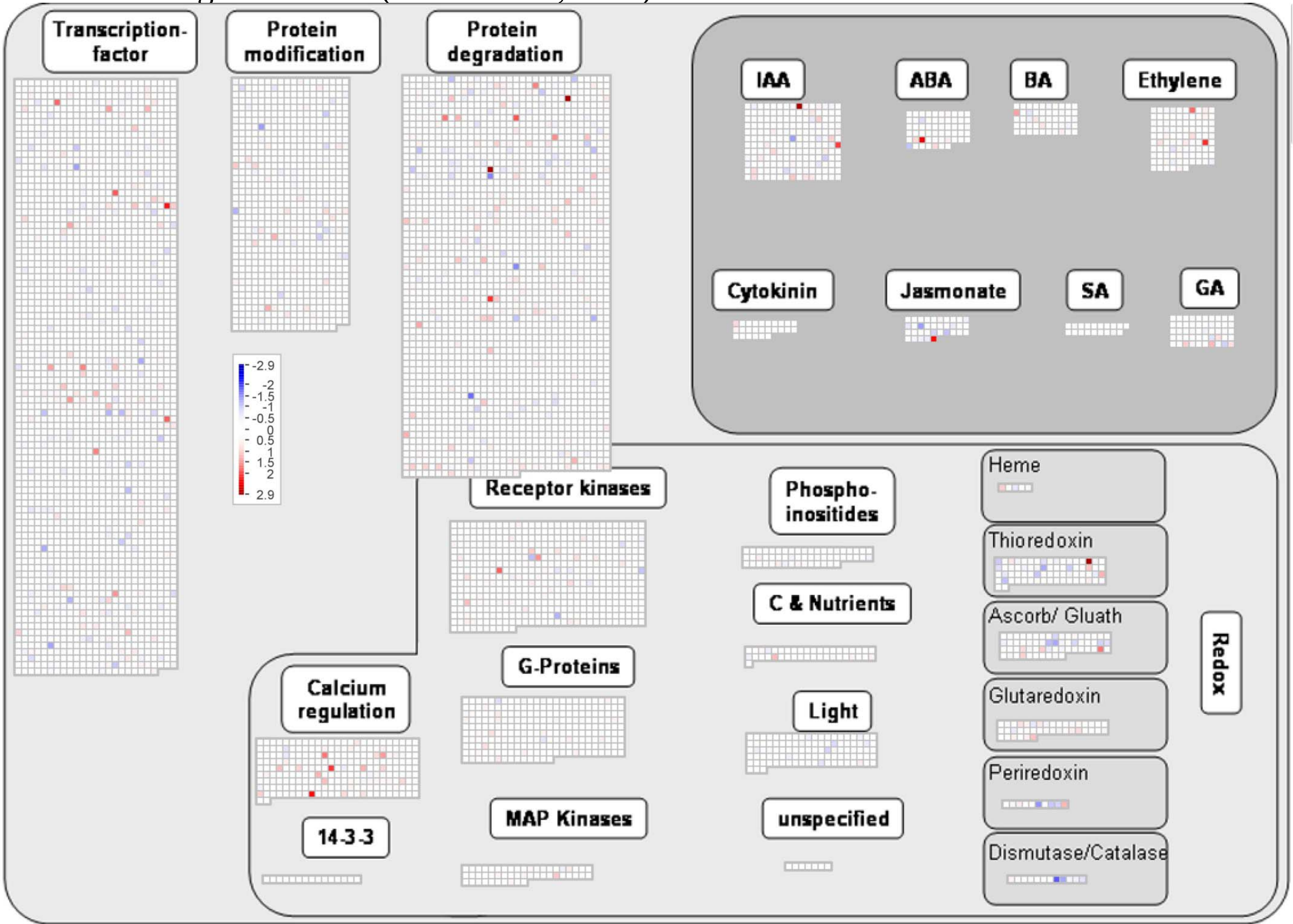

Supplementary Figure S12

A B

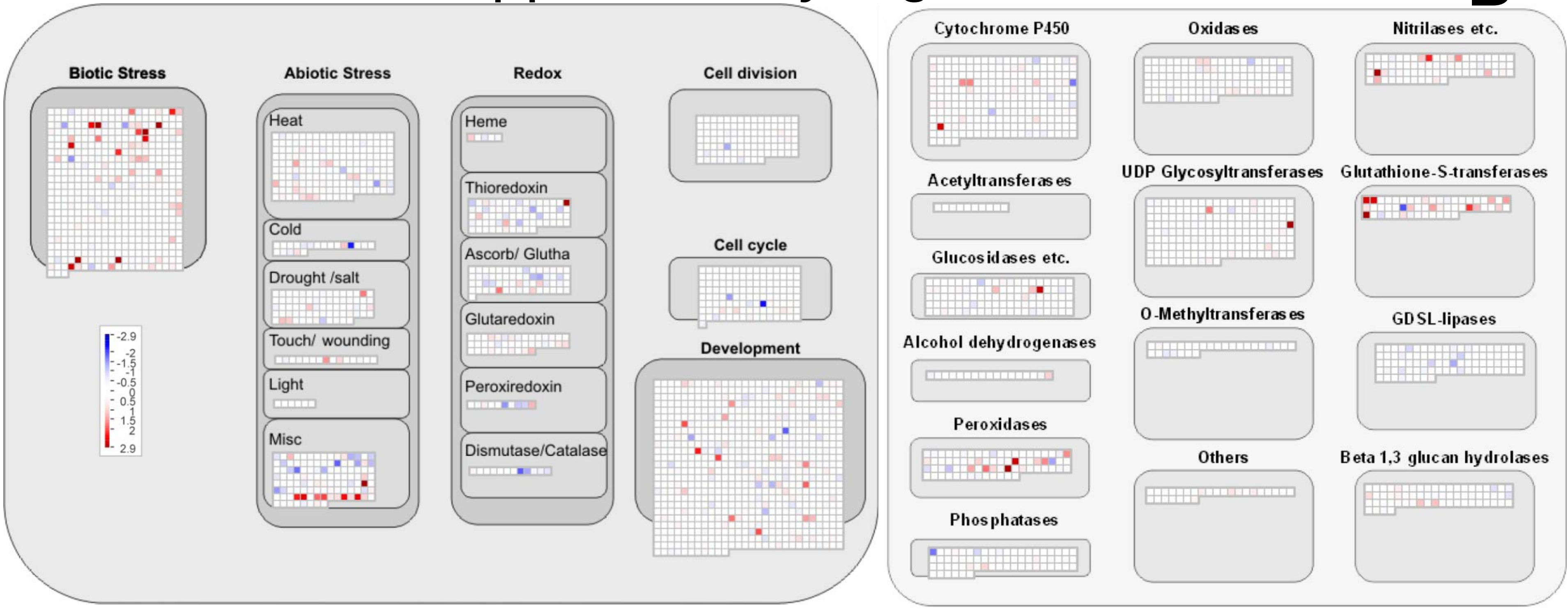

C

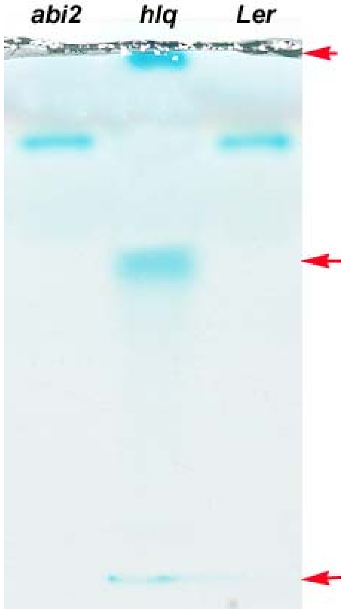

Fig. S12. Transcriptome profiling of *hlq* seedlings to (A) ‘Cellular Response Overview’ and (B) ‘Large Enzyme Families’ pathways using MAPMAN (Usadel et al., 2005). (C) Cell-wall peroxidase isozyme analysis in *hlq*. The cell-wall extract was run on a native-PAGE and submerged in a solution of guaiacol substrate solution. The *hlq* showed the presence of three extra isozymes (red arrows) but not the one present in the wild-type and parental type (*Ler* and *abi2*, respectively). Not equal quantities of total protein were loaded.

Supplementary Figure S13: Transcriptome profiling of *hlq* seedlings to specific families of transcription factors using MAPMAN (Usadel et al., 2005).

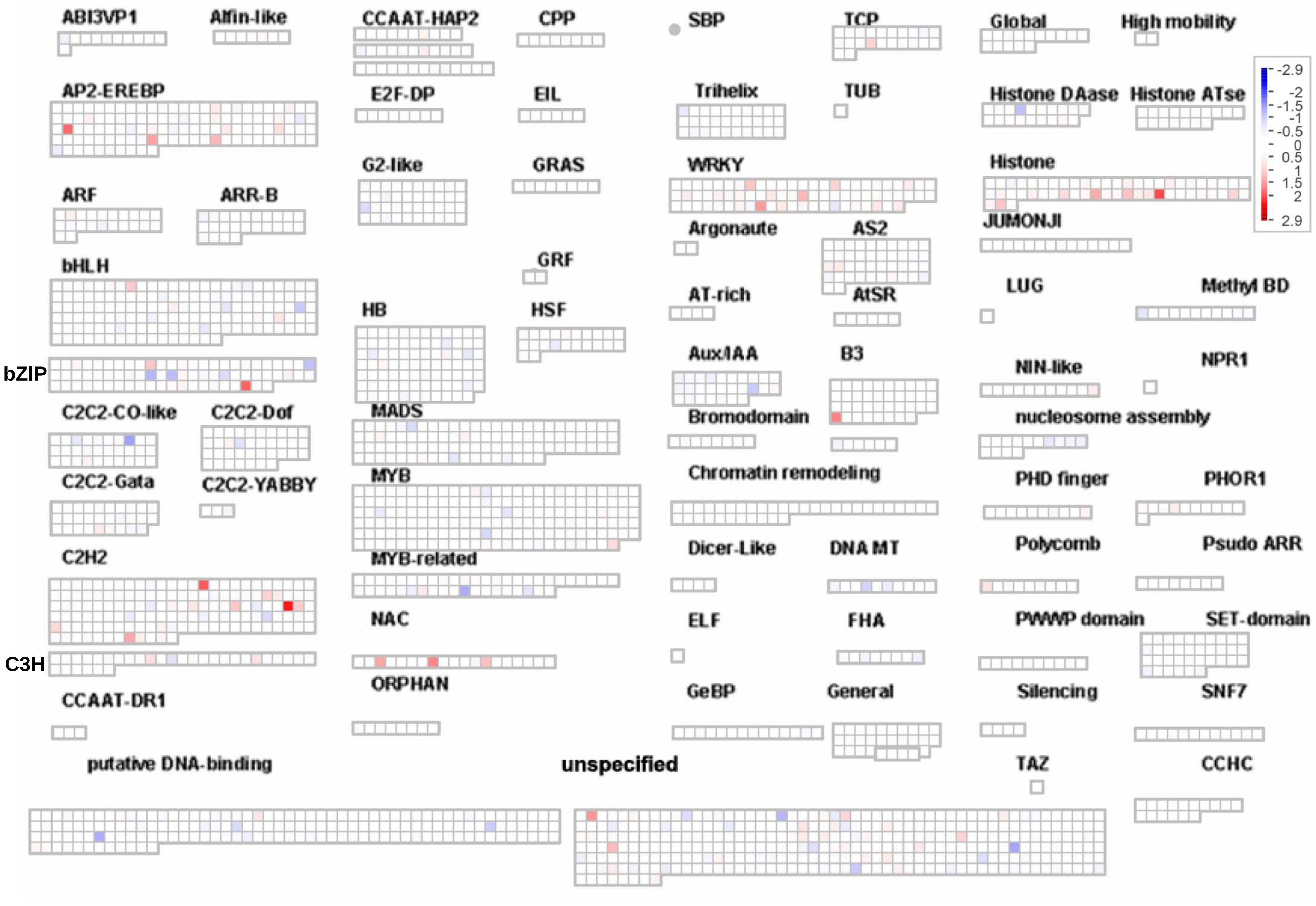

Supplementary Table S1. Cell length parameters of wild type and *hlq* mutant hypocotyl and roots.

**Parameter**	***Ler***	***hlq***
Hypocotyl Epidermal Cell length (µm)	268 ± 47	87 ± 5
Root Epidermal Cell length (µm)	268 ± 23	110 ± 20

Cell length measurements were taken from scanning electron micrographs. Data represent mean ± SD of measurements from 10-15 different cells from 4 or 5 hypocotyls and roots.

Supplementary Table S4. List of *HISTONE, WRKY*, and *AUX/IAA* genes significantly mis-regulated in *hlq* mutant

AGI	Description	*hlq*/WT(log_2)	*p*-value
**Class: Histones**			
At5g10980	histone H3	1.96	0.0001
At1g75600	histone H3.2, putative	1.34	0.02
At4g40040	histone H3.2	1.14	0.0002
At5g59690	histone H4	1.11	0.01
At1g09200	histone H3	0.86	0.0008
At5g10400	histone H3	0.71	0.003
At3g53650	histone H2B, putative	0.58	0.006
At4g27230	HTA2	0.57	0.003
At3g20670	HTA13	0.54	0.02
At2g37470	histone H2B, putative	0.54	0.003
At3g53730	histone H4	0.49	0.009
At1g51060	HTA10	0.47	0.003
At1g19890	ATMGH3	0.45	0.02
At3g27360	histone H3	0.44	0.02
At1g07660	histone H4	0.36	0.02
At3g45980	H2B, HTB9	0.36	0.04
At4g40030	histone H3.2	0.35	0.009
At1g07820	histone H4	0.34	0.02
At3g46320	histone H4	0.31	0.01
At5g59870	HTA6	0.26	0.03
At5g59970	histone H4	0.22	0.04
At5g02560	HTA12	-0.24	0.03
At2g28720	histone H2B, putative	-0.30	0.046
At1g06760	histone H1, putative	-0.47	0.006
**Class: WRKY Transcription Factors**			
At5g13080	WRKY75	1.45	0.0003
At3g56400	WRKY70	1.23	0.0003
At1g64000	WRKY56	1.10	0.0002
At1g80840	WRKY40	0.97	0.001
At5g22570	WRKY38	0.69	0.001
At3g01970	WRKY45	0.69	0.002
At5g64810	WRKY51	0.63	0.0009
At2g30250	WRKY25	0.62	0.01
At2g38470	WRKY33	0.61	0.0008
At2g44745	WRKY12	0.55	0.002
At5g01900	WRKY62	0.51	0.01
At5g26170	WRKY50	0.48	0.02
At2g46400	WRKY46	0.41	0.03
At5g46350	WRKY8	0.41	0.01
At1g62300	WRKY6	0.41	0.04
At2g23320	WRKY15	0.35	0.01
At3g56390	WRKY55-Like	0.32	0.009
At1g29860	WRKY71	0.28	0.02
At2g30590	WRKY21	0.28	0.02
At4g31800	WRKY18	-0.35	0.03
At2g46130	WRKY43	-0.37	0.008
At5g07100	WRKY26	-0.42	0.004
**Class: Aux/IAA Transcription Factors**			
At3g23050	IAA7 /AUXIN RESISTANT2/AXR2	-1.05	0.001
At1g04550	IAA12 /BODENLOS	-0.54	0.01
At1g04100	IAA10	-0.50	0.003
At2g22670	IAA8	-0.44	0.005
At4g28640	IAA11	-0.38	0.006
At5g43700	IAA4/AUX2-11	-0.37	0.03
At1g04240	IAA3/SHY2 (SHORT HYPOCOTYL 2)	-0.35	0.008
At1g52830	IAA6/SHY1	-0.32	0.008
At1g04250	IAA17/AUXIN RESISTANT 3/AXR3	-0.29	0.02
At4g14550	IAA14 /SOLITARY ROOT/SLR	0.39	0.01

Supplementary Table S6. Primers for new markers developed during fine-mapping the *hlq* mutant, and other primers

Marker name	Chr3 (kb)*	Left_Primer	Right_Primer	Anneal Temp.	Prod. size
MIG5 (17)	6087	CGCGCATAATCACAAATCAG	CATCCGAATGCCATTGTTC	50	C<L
MVE11(2)b	6461	TGTAAAGCAAAAACGGATAAAGT	TCCCAAAGTTAAACAAAAGCAAA	55	C>L
T31J18-B	6775	TTGTTGTTTTGTTTCTCCTACATGA	ATTTGTTCCCCTGCTTTTCA	47	C>L
MLD14(1D)	6704	GCCATTTCAAAAAGGATCGAA	TGATTCGTTGGTCGAGTTCA	55	C>L
MPN9a(14)	6940	TTTGCGGTCCCAACATTATT	TGGGATCAGTGTAAAAAGTGACA	55	C>L
MZE19	6962	ACGAGCTTCAGCTTCTCTGC	CCAGATCGAAGTCACGTTCC	55	C>L
CER455083	7042	CAGAAAAAAAAAAACAGAAC	GACGAAACTCACACCATCAC	55	C>L
MQC12(2)b	7094	CACTCACGTCTCCCACCTTT	TTCCAAATACCCAAAACCTTG	47	C<L
K10D20e	7194	AGATTGAATTTTCCAAACAAAGTT	TTCACACGTTTTCGTTCTCCT	47	C>L
F3H11(104)	7211	CAGCAGAACCACCAGATTCA	CTCGTGTGGATCCTCATCATT	55	C>L
MOE17(2)c	7293	CAAGGTTGAAAAGAGAGACAAAG	TTGTGTGTCTTCAGTTTGTTATGA	55	C<L
MFD22(57)	7325	TGTCGAGGCAGAGGATAACA	TCAATTCTGTATTATCGATGATGTG	55	C<L
MSA6(2)	7380	GAATCGGCAAGAGAAGGAGA	CCGTCTTGGAGATTGGAAAC	53	C>L
MXL8	7447	TGATGCTCTTGCAATTGGTC	CTGAACACAAAACCATTGGG	50	C>L
MKA23(1b)	7794	TCCATAACTCGGAATCTCTGAT	CATCCACGTAAAATGAATCTAGC	55	C<L
MKA23(1D)	7813	TTGCACTATTACATTACCGGTTC	GATGCCCATAAATTGCCCTA	55	C<L

MKA23(1E)	7813	GAAGAAAACAGAGCCAATAAAGG	TGACGGGTTTGATTTTGTGA	55	C>L
MXC7(2A)	8234	CAATTCGATCGTCATTACAGAGTT	GGAATGTCATGTATGTATCTCTTGG	55	C>L
K14B15(3A)	8301	CGACCGCAAGAAAATAGCAT	AAGATGATAGTGGTGTGAAGAAAA	55	C<L
MLM24(3G)	8414	GTGCAGCTGGCTTCAAGACT	ACCGAGTGCAATTACGTTCC	55	C>L
MLD14	6748	CACTGCATTGTTGTGGAAAA	CGGGTTGTCAAAGCAAAGTT	55	C>L
MMB12_Taq1[$]	6844	GAATCTTCTCAAACTGAAATCCACC	TCGAAAGGAAGATCGGTGAACC	55	C-Cleaved
MPN9_ACC1[$]	6867	GTGCTATGGTTCAGGAGTTC	CTTACCAGCCATGACGATTC	55	C-Cleaved
MPN9_ALU1[$]	6930	CGGTCATATGCTGGCTGAAG	GACAGCACACAAGTTCCAGG	55	L-Cleaved
MAL21	7041	GCAAAACGGATGAAGAAAGG	TTGGGCTAGTGATGGTGTGA	55	C>L
MQC12	7100	TGAATTTTGTTCCTCTTCAGTCC	GAAGAATGCATCCTACAGTTTCG	55	C>L
k10d20	7166	GGCCAAAAGAGACCAGATTC	TGTAATATGGCAAAAGGGTTTGT	55	C>L
k10d20_HIND3[$]	7188	GGACCTGCCTTTCCCATATC	GCCCAAGCCTCAAGATGTTG	55	C-Cleaved
F3H11	7213	TCATGATGATCTCCCATAGCTT	AAGATCCAACATCAATACACACAC	55	L>C
F3H11_BsmA1[$]	7238	CTGATGGCACTCTTGAACGA	AGAAGATGCTCCACGACACC	55	C-Cleaved
MOE17_Mbo1[$]	7264	TTTGTCTTCAGGCTCATGTTG	GTTTCGTCATCATCGACAACT	55	C>L
MOE17_Alu1[@]	7294	CCTCACAACCCATGAACATGAGAAATTTAGC	TGTATTGGTTACTTAATAGAAATGGCTTT	55	C>L

MFD22	7325	CCAAGGGAATCCAATGAAGC	CAATTCTGTATTATCGATGATGTG	55	L>C
MSA6	7405	CTGGGGTGTTCTCACAGGAT	TTGCCTCTGTGGCTGCTACT	55	C>L
MXL8	7442	CAGGAACAGAGCCAGGAGTT	AAGTAGCCCAAAGCCGTACA	55	C>L
HLQ_int7_flank _RT_F4 (“PF”) HLQ_int7_flank _RT_R4 (“PR”)		TGGAAAGTTTGGTCTGGGTGCAA	GATGACGAGATCTCTATGGGGAGACC	63	55 bp WT 355 bp *hlq*
At3g20780 cDNA primers ; RNA blot		TCGCGGAGAACAAGAACATTGC	AAGGCCGTCCTGGTGCAACAT	63	1342 bp
* TAIR10 $ - CAPS @-dCAPS					
